# Single‐cell deconstruction of post‐sepsis skeletal muscle and adipose tissue microenvironments

**DOI:** 10.1002/jcsm.12596

**Published:** 2020-07-08

**Authors:** Dong Seong Cho, Rebecca E. Schmitt, Aneesha Dasgupta, Alexandra M. Ducharme, Jason D. Doles

**Affiliations:** ^1^ Department of Biochemistry and Molecular Biology Mayo Clinic Rochester MN USA

**Keywords:** Sepsis, Single‐cell RNA‐sequencing, Skeletal muscle, Fat

## Abstract

**Background:**

Persistent loss of skeletal muscle mass and function as well as altered fat metabolism are frequently observed in severe sepsis survivors. Studies examining sepsis‐associated tissue dysfunction from the perspective of the tissue microenvironment are scarce. In this study, we comprehensively assessed transcriptional changes in muscle and fat at single‐cell resolution following experimental sepsis induction.

**Methods:**

Skeletal muscle and visceral white adipose tissue from control mice or mice 1 day or 1 month following faecal slurry‐induced sepsis were used. Single cells were mechanically and enzymatically prepared from whole tissue, and viable cells were further isolated by fluorescence activated cell sorting. Droplet‐based single‐cell RNA‐sequencing (scRNA‐seq; 10× Genomics) was used to generate single‐cell gene expression profiles of thousands of muscle and fat‐resident cells. Bioinformatics analyses were performed to identify and compare individual cell populations in both tissues.

**Results:**

In skeletal muscle, scRNA‐seq analysis classified 1438 single cells into myocytes, endothelial cells, fibroblasts, mesenchymal stem cells, macrophages, neutrophils, T‐cells, B‐cells, and dendritic cells. In adipose tissue, scRNA‐seq analysis classified 2281 single cells into adipose stem cells, preadipocytes, endothelial cells, fibroblasts, macrophages, dendritic cells, B‐cells, T‐cells, NK cells, and gamma delta T‐cells. One day post‐sepsis, the proportion of most non‐immune cell populations was decreased, while immune cell populations, particularly neutrophils and macrophages, were highly enriched. Proportional changes of endothelial cells, neutrophils, and macrophages were validated using faecal slurry and cecal ligation and puncture models. At 1 month post‐sepsis, we observed persistent enrichment/depletion of cell populations and further uncovered a cell‐type and tissue‐specific ability to return to a baseline transcriptomic state. Differential gene expression analyses revealed key genes and pathways altered in post‐sepsis muscle and fat and highlighted the engagement of infection/inflammation and tissue damage signalling. Finally, regulator analysis identified gonadotropin‐releasing hormone and Bay 11‐7082 as targets/compounds that we show can reduce sepsis‐associated loss of lean or fat mass.

**Conclusions:**

These data demonstrate persistent post‐sepsis muscle and adipose tissue disruption at the single‐cell level and highlight opportunities to combat long‐term post‐sepsis tissue wasting using bioinformatics‐guided therapeutic interventions.

## Introduction

Despite advances in critical/intensive care, sepsis remains a leading cause of death worldwide. While the short‐term mortality associated with sepsis is improving, the number of sepsis survivors with chronic illness is rapidly increasing.^1^, ^2^ These sepsis survivors often experience a higher risk of hospital readmissions, increased infection susceptibility, skeletal muscle dysfunction, cardiovascular disease, and cognitive impairment—and even death—for years following a sepsis episode. In the first year alone, approximately 60% of sepsis survivors have at least one re‐hospitalization visit, often due to infection.^3^, ^4^ One year mortality rates of ~15% underscore the severity of the long‐term issues facing sepsis survivors.^5^ Given the dearth of interventions to limit long‐term tissue/organ dysfunction, there is a need to better understand basic mechanisms of post‐sepsis organ dysfunction in order to improve sepsis survivorship.

Skeletal muscle and adipose tissue are two key metabolic tissues that exhibit major functional and histological changes in sepsis survivors.^6^ Analogous to cachexia syndrome, muscle and fat wasting post‐sepsis can result in many detrimental outcomes that directly impact long‐term mortality and quality of life including decreased locomotive capacity, respiratory dysfunction, and altered systemic metabolism.^7^, ^8^ Fundamentally, tissue homeostasis involves balancing pro‐growth anabolic processes with breakdown‐associated catabolism. In the context of sepsis, acute loss of muscle and fat mass often occurs due to hyper‐catabolism linked to infection‐associated inflammation, decreased nutrient intake, disuse, or altered systemic metabolism.^9^, ^10^ An inability to replenish lost tissue mass post‐sepsis can then result as a consequence of blunted hypertrophy, suppressed stem activity/tissue regeneration capacity, and persistent macromolecule catabolism. While mechanisms of persistent muscle/fat dysfunction are incompletely understood, several key players are known. For example, mitochondria damage/defects may contribute to long‐term muscle stem/satellite cell dysfunction, thus compromising muscle regeneration.^11^ Additionally, oxidative stress frequently observed post‐sepsis may contribute to tissue wasting via multiple mechanisms including enhanced proteolytic pathway (caspases and calpains) activity, deregulated ubiquitin proteasome system function, enhanced autophagy, and suppressed anabolic pathway activity.^12^, ^13^ Unfortunately, efforts targeting these pathways in cellular and pre‐clinical models have yielded mixed results, highlighting the need to identify new targets, including those that may aid in repletion of lost tissue mass.

Tissue repair following damage/atrophy is a coordinated process involving many specialized cell types. In skeletal muscle, the local mononuclear cell environment is a complex population of muscle stem cells, fibroblasts/fibro‐adipogenic progenitor cells, endothelial, immune (B‐cells and T‐cells, macrophages, and neutrophils) cells, and a few minor cell populations such as tenocyte precursors and pericytes.^14^ Upon injury, each of these cell populations contributes to the repair/regeneration process. Indeed, loss or precocious expansion of any of these cell types often impedes timely tissue repair or is sufficient to drive tissue atrophy. Examples of non‐muscle cell contributions to skeletal muscle repair include connective tissue fibroblasts and macrophages, both of which can influence multiple aspects of myogenesis such as satellite cell activation, myoblast expansion, and terminal differentiation.^15^, ^16^ While there are numerous examples documenting changes in many of these individual cell populations in post‐sepsis muscle and fat,^8^, ^11^, ^17^ comprehensive analyses are lacking.

Single‐cell transcript and protein profiling involves simultaneous biochemical evaluation of hundreds/thousands of cells in a single sample, permitting highly sophisticated views into the heterogeneity of complex tissues and diverse cell populations. In particular, droplet‐based single‐cell mRNA‐sequencing (scRNA‐seq) has been used to query tissue heterogeneity in myriad biological contexts including development, aging, disease, and cancer, and in experimental models including but not limited to yeast, drosophila, zebrafish, mice, and humans.^18^ In this study, we leverage scRNA‐seq to profile alterations in murine muscle and fat microenvironments in response to a severe infection/experimental sepsis. Our data show widespread changes in multiple tissue‐resident cell populations acutely following infection and reveal persistent alterations in selected cell populations 1 month following infection. Combined bioinformatics analyses of both tissues provide insight into tissue resilience/recovery following infection and highlight potential opportunities to target conserved signalling pathways and/or cell populations to limit organ dysfunction in sepsis survivors.

## Methods

### Animals

All animal experimental procedures were approved by the Mayo Clinic Institutional Animal Care and Use Committee. Three‐ to 4‐month‐old male FVB mice and 11‐month‐old female C57Bl/6 mice were used. For the faecal‐induced peritonitis (FIP) model, sepsis was induced on FVB mice using a single 500 μL intraperitoneal (IP) injection of a 50 mg/mL faecal slurry (FS) as previously described.^19^ Briefly, faeces from individual mice were collected, pooled, weighed, and dissolved in 0.9% saline solution. FS was then filtered using a 70 μm cell strainer and immediately injected IP into recipient mice.

The cecal ligation and puncture (CLP) model was performed as previously described^20^, ^21^ with minor modifications. Briefly, C57Bl/6 mice were anaesthetized using an IP injection of ketamine (Ketaset; Ketamine HCL Injection, USP 100 mg/mL)/xylazine (AnaSed; Xylazine 20 mg/mL) dissolved in sterile saline. The cecum was extracted from the abdomen and ligated 1.0 cm from the tip. Two holes were punctured at the end of the cecum with a 25‐G (BD Biosciences) needle, faecal matter released, and the cecum returned to the abdomen. Control mice underwent sham surgery (incision to access abdominal cavity). All mice received 0.5 mg/kg Buprenorphine SR‐LAB (ZooPharm, 1 mg/kg) directly after abdominal closure. Two hours post‐surgery, CLP mice received antibiotics [25 mg/kg of imipenem monohydrate (LKT Labs)] diluted in 1 mL saline subcutaneously while control mice received 1 mL saline. Antibiotics or saline injections were continued twice per day, for 72 h, for each respective group.

For drug/treatment studies, 3 days after FIP, mice were separately administered the following chemicals: 5‐fluorouracil (10.5 mg/mL) (Sigma‐Aldrich), valproic acid (60 mg/mL) (Sigma‐Aldrich), gonadotropin‐releasing hormone (GnRH) (0.03 mg/mL) (Sigma‐Aldrich), and Bay 11‐7082 (6 mg/mL) (Sigma‐Aldrich) dissolved in 100 μL 0.9% saline solution. Treatment was performed by IP injection daily for five consecutive days. Drug concentrations were selected based on previous studies.^22^, ^23^, ^24^, ^25^ Lean mass and fat mass were measured using EchoMRI body composition analysis (Echo Medical Systems, Houston, Texas).

### Single‐cell isolation

Tibialis anterior (TA) muscle and epididymal fat pads were dissected from mice in each experimental condition. Tissues were enzymatically dissociated as described previously.^26^ Briefly, muscle tissues were minced and dissociated in 0.2% Collagenase II (ThermoFisher) for 1 h in 37 °C. Dissociated cells from muscle tissues were then filtered through 40 μm cell strainer. Epididymal fat pads were minced and digested in 0.1% Collagenase II for 1 h in 37 °C. Dissociated cells from fat pads were then filtered through 70 μm cell strainer.

Isolated cells from muscle and adipose tissues were stained with propidium iodide solution (1:100) (Miltenyi Biotec). Then, live (propidium iodide^neg^) single cells were isolated by fluorescence‐activated cell sorting with indicated gating strategies at the Mayo Clinic Microscopy Cell Analysis Core Flow Cytometry Facility (Rochester, MN, USA).

### Single‐cell RNA‐sequencing and data processing

Freshly sorted single cells from muscle and adipose tissues from a pool of three mice for each condition were isolated as described previously. Cells were captured, and complementary DNA libraries were prepared for scRNA‐seq using Chromium Single Cell 3′ Reagent Kit v2 (10× Genomics). Complementary DNA libraries were sequenced on an Illumina HiSeq 4000 with paired‐end 100 bp reads. All of these scRNA‐seq procedures were performed in collaboration with Mayo Clinic Medical Genome Facility (Rochester, MN, USA). Sequencing outputs were processed by Cell Ranger 2.2.0 (10× Genomics) to align to the mm10 genome and generate unique molecular identifier counts in collaboration with Mayo Clinic Bioinformatics Core (Rochester, MN, USA).

Data were analysed using Seurat package (Version 2.3.4)^27^, ^28^ in RStudio software (Version 1.1.456). To remove cells with poor quality, cells with high fraction of mitochondrial transcripts (10% and 5% for muscle and adipose tissue, respectively), with low unique molecular identifier counts (1000 and 2000 for muscle and adipose tissue, respectively), and with low number of detected genes (500) were removed for analysis. Data were normalized using NormalizeData function.

### ScRNA‐seq data analysis

Principal component analysis (PCA) analysis was performed using RunPCA function on the variable genes of each sample identified by FindVariableGenes function. Cells were then projected and visualized on two‐dimensional maps with uniform manifold approximation and projection (UMAP).^29^ Clusters for control samples were identified using FindClusters function based on shared nearest neighbour‐based clustering on the first 10 principal components. Differentially expressed genes (DEGs) in each cluster were found by FindMarkers function. All of these functions (FindVariableGenes, FindClusters, and FindMarkers) are included in Seurat package (Version 2.3.4). DEGs were used to define each cluster into a specific cell type based on cell‐type markers reported in PanglaoDB database^30^ (please see Supporting Information, *Figures* *S2* and *S6*, for full list of markers in each cluster). For clusters that were classified into same cell type and that were in distinct areas in UMAP projections (i.e. macrophage in muscle dataset), DEGs were identified between these clusters, and the clusters were further classified into more specific cell type.

Cell‐type classification of control samples was then used to classify cells of 1 day and 1 month post‐sepsis samples using BuildRFClassifier and ClassifyCells functions of Seurat package (Version 2.3.4). DEGs for 1 day and 1 month post‐sepsis samples were identified as described earlier and were used to assess cell‐type classification. For clusters that are subdivided into multiple groups in UMAP projection, the clusters were separately reclustered using FindClusters function. DEGs for these reclustered subsets were identified, and cell types were defined similarly as described previously. Cell‐type specific DE genes in comparison to control were identified using FindMarkers. Pathway analysis and upstream regulator analysis was performed on the DEGs (fold change >1.5, *P* < 0.01, and adjusted *P* < 0.05) with Ingenuity Pathway Analysis software (QIAGEN).

### Immunohistochemistry

Tibialis anterior (TA) muscle was frozen in optimum cutting temperature compound (Tissue‐Tek) and sectioned in 8 μm slices. Tissue sections were fixed in 4% paraformaldehyde for 10 min at room temperature. Fixed tissue sections were permeabilized with 0.2% Triton X‐100 in phosphate buffered saline, followed by blocking with 3% bovine serum albumin, 0.2% Triton X‐100, and 0.2% Tween 20 in phosphate buffered saline for 30 min. Tissue sections were then stained with the following primary antibodies in 4 °C overnight: anti‐CD31 (1:50) (Miltenyi Biotec, 130‐102‐519), anti‐HDC (1:80) (LSBio, LS‐C334075), and anti‐CD11b‐APC antibodies (1:50) (Biolegend, 101211). Secondary antibody incubations were performed at room temperature for 30 min for CD31 and HDC staining with goat anti‐rat Alexa 647 antibody (1:500) (ThermoFisher) and goat anti‐rabbit Texas Red antibody (1:500) (ThermoFisher), respectively. Tissue sections were then incubated with 1 μg/mL DAPI for 10 min. Cells positive for each marker were quantified in at least six different fields (~11 mm^2^) for each replicate.

## Results

### Single‐cell analyses reveal multiple distinct tissue‐resident cell populations in murine skeletal muscle and visceral white adipose tissue

**Figure 1:**
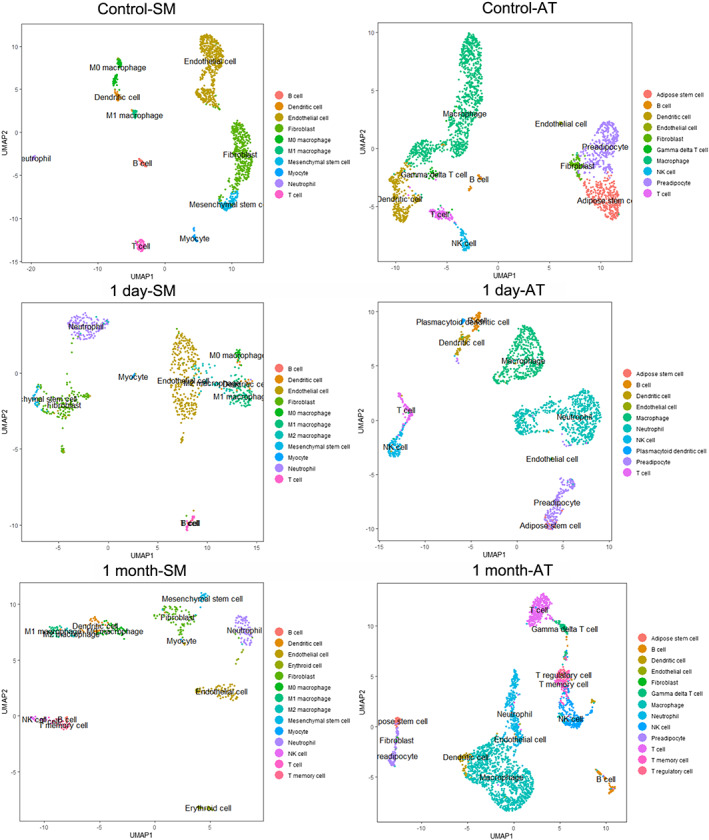
Single‐cell RNA‐sequencing analysis of murine skeletal muscle (SM) and adipose tissues (AT) after faecal slurry (FS)‐induced sepsis (FIP). Uniform manifold approximation and projection (UMAP) of single cells dissociated from tibialis anterior (TA) muscle (left panels) and from epididymal fat pads (right panels). Single cells were classified into 10 distinct cell populations in control conditions (top row). UMAP projections of single cells from TA muscle and epididymal fat pads after 1 day (centre row) and 1 month (bottom row) post‐sepsis are shown. Detailed analyses of population identification and classification can be found in the Supporting Information and Methods section.

The first objective of this study was to establish a baseline single‐cell landscape of normal, murine skeletal muscle. We chose to profile tibialis anterior (TA) muscle in this study as the TA (and other hindlimb muscles) is one of the most widely studied skeletal muscles in mice. Pooled TA muscle from control (healthy) male FVB mice was enzymatically digested, and viable single cells from pooled tissue suspensions were isolated by fluorescence‐activated cell sorting sorting (Supporting Information, *Figure* *S1* *A*). Single viable cells were profiled using the 10× Genomics droplet‐based scRNA‐sequencing platform, resulting in the generation of 1619 single‐cell transcriptomes. After removal of cells with transcriptomes of subpar sample quality, 1438 cells were retained for further analysis. Initial visualization and two‐dimensional mapping were performed using UMAP^29^ (Supporting Information, *Figure* *S1* *B*). Single cells were then grouped into 18 clusters that captured all distinct clusters in the UMAP projection. Based on the DEGs in each cluster, population identities were then assigned based on markers reported in PanglaoDB database^30^ (*Figure* 1, and Supporting Information, *Figures* *S2*‑*S4*; please see Supporting Information, *Figures* *S2* and *S3*, for full list of markers in each cell type). Following cluster identification, redundant cell populations were consolidated resulting in the clear delineation of 10 main cell populations. These analyses thus defined the baseline mononuclear muscle microenvironment as comprising 80% non‐immune cells (37% endothelial cells, 31% fibroblasts, 9% mesenchymal stem cells, and 3% myocytes) and 20% immune cells [3% B‐cells, 3% dendritic cells, 5% uncommitted (M0) macrophage, 2% M1‐type macrophage, 2% neutrophils, and 5% T‐cells].

In parallel, scRNA‐seq was performed on 2,396 viable single cells from epididymal fat pads of control male FVB mice, followed by removal of cells with subpar transcriptome quality, resulting in 2,281 high quality single‐cell transcriptomes for downstream analysis (*Figure* 1and Supporting Information, *Figures* *S5*‑*S8*). Ten cell populations in the adipose tissue microenvironment were then defined: 34% non‐immune cells (15% adipose stem cells, 15% preadipocytes, 3% fibroblasts, and 1% endothelial cells) and 66% immune cells (42% macrophages, 12% dendritic cells, 4% NK cells, 4% T‐cells, 2% B‐cells, and 2% gamma delta T‐cells; please see Supporting Information, *Figures* *S6* and *S7*, for full list of markers in each cell type). Together, these data highlight the cellular complexity of skeletal muscle and visceral white adipose tissue under healthy, baseline conditions.

### Sepsis induces significant changes in muscle and adipose tissue mononuclear cell composition

We next induced experimental sepsis in FVB mice by IP injection of a FS/FIP^19^ to investigate the acute and long‐term effects of severe infection on cellular heterogeneity in muscle and adipose tissue. Twenty‐four hours following sepsis‐induced infection, total body, and fat pad wet weights exhibited slight but insignificant (*P* > 0.05) decreases compared with control, sham‐injected mice whereas TA weight was significantly (~15% reduction) lower (Supporting Information, *Figure* *S9*). At 1 month following FS injection/FIP, total, TA, and fat pad weights were all significantly decreased (~10%, ~30%, and ~85%, respectively), with adipose tissue exhibiting a particularly large (~six‐fold) reduction in mass (Supporting Information, *Figure* *S9* *C*).

Single‐cell RNA‐sequencing (scRNA‐seq) was then performed on single cells from TA muscle and epididymal fat pads of mice 1 day (1D) and 1 month (1M) post‐sepsis/FS/FIP (*Figure* 1 and Supporting Information, *Figures* *S10*‑*S15*). Using the classification scheme defined in control samples, cell populations in muscle and fat were determined for each condition (see Methods section for analysis details; *Figure* 1 and Supporting Information, *Figures* *S10‑S15*). While most post‐sepsis populations were well clustered into cell types based on control‐defined marker expression, others required further analyses. Ambiguously defined cell populations were identified as follows: M1 macrophages in muscle 1D and 1M, macrophages and B‐cells in fat 1D and 1M, and T‐cells in fat 1M samples (Supporting Information, *Figures* *S10*, *S11*, *S13*, and *S14*). As these data comprise cell types that were not identified in control samples, these new populations were further subclustered and defined as follows: (i) M1 macrophage (muscle 1D and 1M) into M1 and M2 macrophages; (ii) endothelial cells (muscle 1M) into endothelial cells and erythroid cells; (iii) fibroblasts (muscle 1M) into fibroblasts and mesenchymal stem cells; (iv) T‐cells (muscle 1M) into T‐cells, NK cells, and T‐memory cells; (v) macrophages (fat 1D and 1M) into macrophages and neutrophils; (vi) B‐cell (fat 1D) into B‐cell, neutrophils, and plasmacytoid dendritic cells; (vii) B‐cells (fat 1M) into B‐cells and neutrophils; and (viii) T‐cells (fat 1M) into T‐cells, gamma delta T‐cells, T‐memory cells, and T‐regulatory cells (*Figure* 1).

We then combined individual UMAP projections (control, 1D, and 1M) into a single UMAP projection for each tissue in order to directly compare all three experimental conditions (Supporting Information, *Figures* *S12* and *S15*). Combined UMAP projections of both tissues show that while individual cell‐type classifications between conditions are similar, that many cell populations in 1D and 1M samples cluster away from their control counterparts (Supporting Information, *Figures* *S12* and *S15*). These data suggest that while cellular identity is not compromised by severe infection, that significant phenotype alterations exist in selected cell populations in acute and long‐term post‐sepsis tissues.

**Figure 2:**
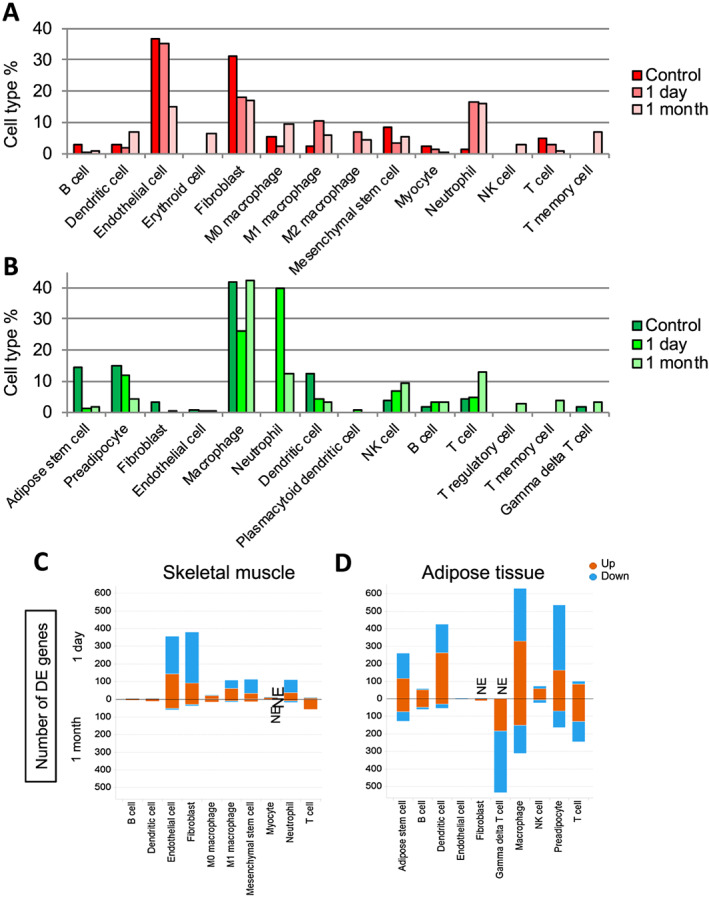
Comparative analyses of post‐sepsis skeletal muscle‐resident and adipose tissue‐resident cell population abundance and gene expression changes. (A, B) Bar graphs depicting the frequency of each cell type in each sample in skeletal muscle (A) and adipose tissue (B). (C, D) Graphs depicting the number of differentially expressed genes in each cell type compared with control in muscle (C) and adipose tissue (D). Orange = up‐regulated transcripts, and blue = down‐regulated transcripts. NE: cells do not exist.

Quantification of individual cell types revealed notable changes in both muscle and fat post‐sepsis. 1D post‐sepsis muscle was composed of fewer fibroblast and mesenchymal stem‐like cells whereas M1/M2 macrophage and neutrophil cells were more abundant (*Figure* 2A). At the 1M timepoint, endothelial cell number was dramatically reduced, and the local immune microenvironment exhibited increased heterogeneity with appearance of NK and T‐memory cells. Similar to muscle, the acute (1D) post‐sepsis fat microenvironment contained decreased fractions of non‐immune cells (adipose stem cells, preadipocytes, and fibroblasts) while the fraction of neutrophils was dramatically increased (*Figure* 2B). At the 1M timepoint, the adipose microenvironment displayed a more heterogeneous T‐cell repertoire, with appearance of T‐regulatory and T‐memory cells that were both not detected in control and 1D samples.

### Gene expression analyses reveal differential cellular and tissue resilience patterns following severe sepsis

As shown in the combined tissue UMAP projections in Supporting Information, *Figures* S12 (muscle) and *S15* (fat), most cell types appear distinct from their control counterparts 1D post‐sepsis. Some, but not all, of these differences are resolved 1M post‐sepsis. To find cell‐type specific gene expression changes, the populations of each cell type in 1D and 1M samples were separately compared with matched control populations. First, we quantified the number of DEGs (*P* < 0.01, adjusted *P* < 0.05, and fold change >1.5) in each cell type in comparison to control (*Figure* 2C and 2D). In both muscle and fat, most cell types had much higher number of DEGs 1D post‐sepsis than 1M post‐sepsis. In contrast, T‐cells present in both tissues had greater number of DEGs in 1 M post‐sepsis likely driving the heterogeneous clustering of those populations at this timepoint (*Figures* 2). This analysis indicates that most muscle and fat‐resident cell types at 1 M post‐sepsis are more similar to control cells than their 1D counterparts. To corroborate this finding, we performed correlation analyses and found that Pearson correlation coefficients of most 1M cell populations were higher than 1D cell populations when independently compared with controls (Supporting Information, *Figure* S16, above diagonal line). Additionally, the overall number of DEGs was less in muscle compared with fat when combining 1D and 1M comparisons vs. control (*Figure* 2C and 2D). Notably, muscle cell populations 1M post‐sepsis exhibited markedly low numbers of DEGs, indicating that fat‐resident cell populations are less resilient than muscle‐resident cell populations following sepsis.

### Cell‐type specific comparisons identify genes and signalling pathways acutely and persistently altered post‐sepsis

**Figure 3:**
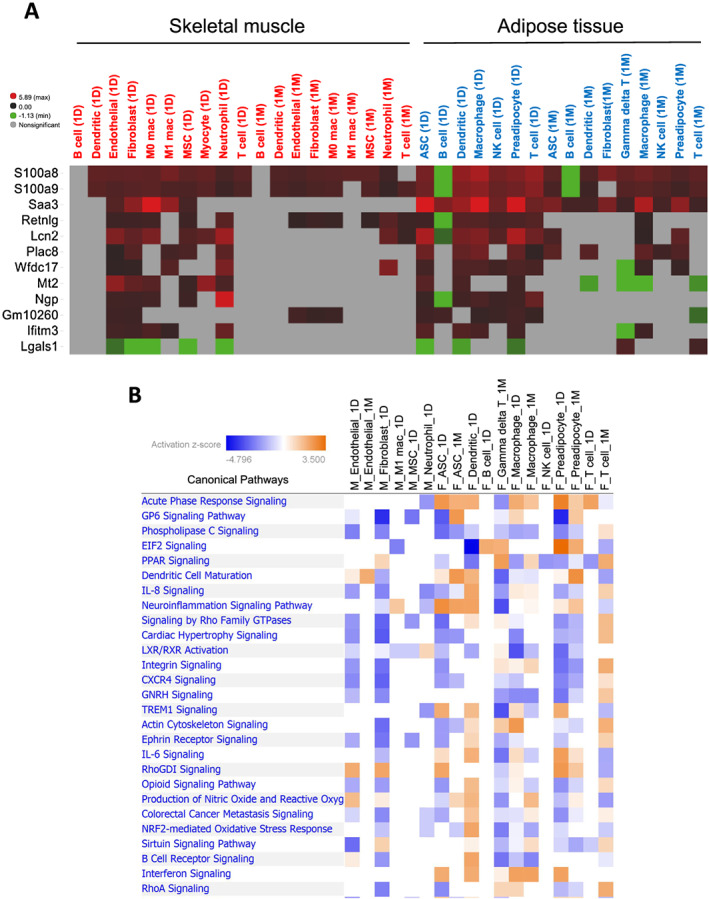
Combined analysis of DE genes in muscle and adipose tissues. (A) A heatmap depicting DE genes in at least five comparisons in both muscle and fat. Log fold changes compared with the same cell type in control were shown with colours. Red = up‐regulated, green = down‐regulated, and grey = no significant difference. (B) A chart depicting top signalling pathways associated with DE genes in muscle and adipose tissues. Cell type/experimental comparisons in muscle vs. adipose tissue are prefaced with ‘M_’ and ‘F_’, respectively. Coloured pathways are statistically significant for a given cell type (*P* < 0.05). Blue = negative pathway association, and orange = positive pathway association.

We next sought to determine patterns of DEGs commonly altered across cell types in each tissue. Among the DEGs within each cell type at 1D and 1M post‐sepsis compared with control, several genes emerged as common DEGs (*Figure* 3A and Supporting Information, *Figures* *S17* and *S18*). In muscle, genes that were significantly different at 1D and 1M in both immune and non‐immune cells compared with controls included S100a8, S100a9, Retnlg, Lcn2, Mt2, Saa3, Ifitm3, and Ngp (Supporting Information, *Figure* S17). In fat, Saa3, S100a8, S100a9, Lcn2, Mt1, and Retnlg were perturbed in the majority of analysed cell types (Supporting Information, *Figure* S18). We then combined the DEGs of muscle and fat to identify common DEGs across tissues (*Figure* 3A). Damage‐associated molecular pattern (DAMP) transcripts S100a8, S100a9, Saa3, and Lcn2 were identified as highly up‐regulated in >50% of all cell types analysed in both tissues. Pathway analyses of common DEGs identified pathways associated with infection, damage response, and inflammation such as acute phase response signalling, EIF2 signalling, IL‐8 signalling, and neuroinflammation signalling pathways (*Figure* 3B). Taken together, these combined cell‐type and cross‐tissue gene expression analyses revealed key post‐sepsis transcriptional alterations that could serve as a guide for pre‐clinical intervention studies.

### Validation of sepsis‐induced cell‐type composition alterations using multiple murine sepsis models

**Figure 4:**
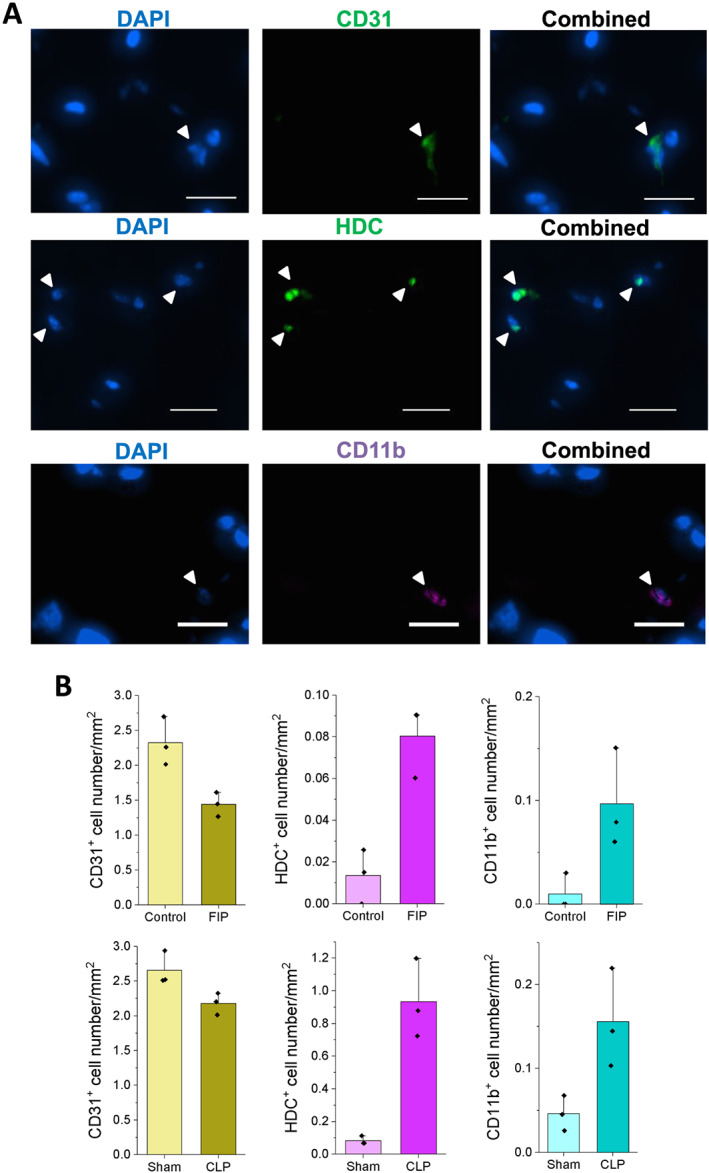
Immunohistochemistry analysis of endothelial cells, neutrophils, and macrophages in faecal slurry/FIP and cecal ligation and puncture (CLP) sepsis models. (A) Representative immunofluorescence images of tibialis anterior muscle sections stained with CD31 (top), HDC (centre), and CD11b (bottom) antibodies. (Scale bars: 100 μm). (B) Quantification of the number of CD31, HDC, or CD11b positive cells (per mm squared) in faecal slurry (top) or CLP models (bottom). Immunohistochemistry staining was performed 19 days after FIP and 16 days after CLP. *P*‐values (Student's *t*‐test) of all pairwise comparisons were less than 0.1.

To validate changes in post‐sepsis cell‐type distribution, we stained TA muscle cross sections from two groups of experimental mice (plus controls): FS/FIP and CLP (*Figure* 4). We used antibodies against CD31, HDC, and CD11b to identify endothelial cells, neutrophils, and macrophages, respectively (*Figure* 4A). In both FIP and CLP models, the numbers of CD31^+^ cells were significantly lower, while HDC^+^ cells (neutrophils) and CD11b^+^ cells (macrophages) were significantly more abundant compared with respective control tissues (*Figure* 4B). These data corroborate changes in endothelial cell, neutrophil, and macrophage population abundance detected in the scRNA‐seq analysis.

### Upstream regulator analysis identifies targets/compounds that may aid in post‐sepsis muscle or fat recovery

**Figure 5:**
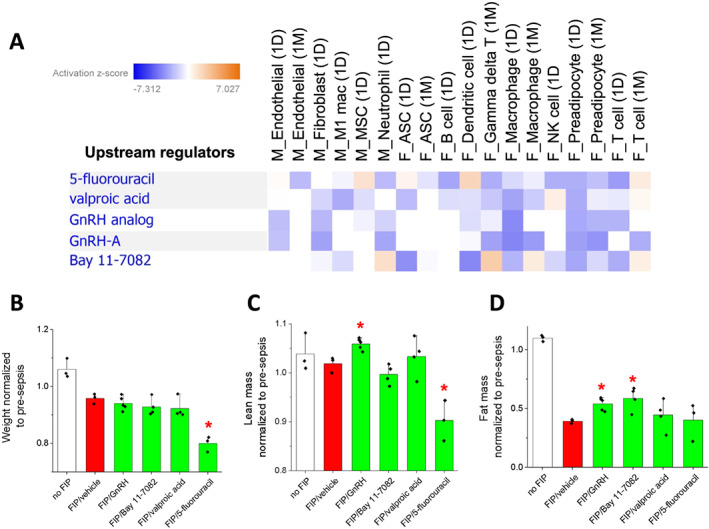
Upstream regulator analysis of post‐sepsis DEGs. (A) A chart depicting upstream regulators predicted to activate or inhibit expression changes in muscle and adipose tissue cell types. Blue = predicted to inhibit expression changes, and orange = predicted to activate expression changes. (B‑D) Graphs depicting weight (B), lean mass (C), and fat mass (D) upon treatment with the identified compounds. All measurements were normalized to pre‐sepsis conditions. A red asterisk indicates a statistically significant difference (*P* < 0.1, Student's *t*‐test) compared with control (FIP/vehicle).

We next performed an upstream regulator analysis on the common DEGs across selected muscle and adipose cell populations to predict molecules that could potentially counteract post‐sepsis gene expression changes (*Figure* 5A and Supporting Information, *Table* S1). Among the predicted upstream regulators, a few molecules including 5‐fluorouracil, valproic acid, GnRH, and Bay 11‐7082 had high negative activation *z*‐scores in the DEGs of the majority of the analysed cell types (please see Supporting Information, *Table* S1, for details of analysis including the DEGs regulated by these molecules). This negative *z*‐score indicates that these molecules are predicted to inhibit observed gene expression changes. As a proof‐of‐principle study, we sought to evaluate the effects of these molecules during post‐sepsis tissue recovery. We separately treated FIP mice with GnRH, Bay 11‐7082, valproic acid, and 5‐fluorouracil by IP injection for five consecutive days starting 3 days after FIP. As expected, FIP induced overall weight, lean, and fat mass loss (*Figure* 5B‑5D). Although drug‐treated mice did not exhibit improvements in overall weight 8 days after FIP, GnRH‐treated mice gained significantly more lean mass than vehicle‐treated mice (*Figure* 5C). Additionally, both GnRH and Bay 11‐7082 treatments significantly increased post‐FIP fat mass (*Figure* 5D). Given the significant and often persistent tissue loss in post‐sepsis mice (Supporting Information, *Figure* S9B and S9C) and patients, these results suggest that single‐cell analyses are a powerful platform from which we can identify and possibly evaluate therapies to counteract long‐term tissue loss.

## Discussion

Our data provide evidence that an acute severe infection can impart lasting changes to local muscle and fat microenvironments. Common cellular alterations observed in both muscle and fat included progenitor cell, endothelial cell, and fibroblast depletion, along with neutrophil and NK cell persistence. These results dovetail with other published reports describing long‐lasting phenotypes associated with persistent inflammatory catabolism syndrome, a syndrome commonly affecting sepsis survivors.^4^, ^31^ In several reports, low‐level chronic elevation of immune cells such as M1‐type macrophages, neutrophils, certain T‐cell subtypes, or myeloid‐derived suppressor cells can be detected in mouse models of sepsis or in human samples^32^, ^33^ in the absence of an overt persistent infection. Given many reports documenting post‐sepsis functional deficits in tissues such as skeletal muscle,^8^ it would be interesting to further explore the relationship between persistent local inflammatory cell populations and tissue maintenance/repair in a post‐infection context.

On a transcript level, common themes among DEGs across cell types/tissues were response to infection and tissue damage. Examples include (i) S100a8, S100a9, and Retnlg in brain^34^, ^35^; (ii) Lcn2 in brain and liver^35^, ^36^; (iii) Mt1 and Mt2 in macrophages, liver, kidney, ovary, lung, uterus, brain, pancreas, heart, spleen, and intestine^37^, ^38^, ^39^, ^40^; (iv) Ifitm3 in T‐memory cells in brain and lung^41^, ^42^; (v) Ngp in brain and lung^35^, ^43^; and (vi) Saa3 in lung and epithelial cells.^44^, ^45^ Notably, one striking finding from our study was the significant upregulation of S100a8/9, Saa3, and Lcn2 across multiple cell types in both muscle and fat (*Figure* 3A and Supporting Information, *Figures* *S17* and *S18*). These transcripts are all linked to DAMP signalling, which, in conjunction with residual pathogen‐associated molecular pattern (PAMP) molecules, may perpetuate a systemic inflammatory response as well as local tissue inflammation in sepsis survivors.^46^ We observed acute (1 day) and chronic (1 month) DAMP transcript upregulation in both immune (dendritic cells, T‐cells, macrophages, and neutrophils) and non‐immune (fibroblasts, endothelial cells, and tissue progenitor cells) suggesting that low‐level tissue damage may drive local inflammation and persistent tissue dysfunction. Considering recent advances targeting specific DAMP molecules^47^, ^48^ or broad classes of pro‐inflammatory DAMPs^49^ to mitigate acute sepsis mortality,^50^ it would be interesting to determine the extent to which DAMP/PAMP reduction would aid post‐sepsis muscle and fat tissue repletion.

In both tissues, we observed the greatest differences in population‐specific differential gene expression 1 day following infection (*Figure* 2C and 2D). In skeletal muscle, the number of DEGs within a given cell population sharply decreased 1 month post‐infection (*Figure* 2C), suggesting that while cell population abundance is altered compared with control muscle, the molecular (transcriptional) state of a given cell population is largely able to return to baseline. It also suggests that altered abundance rather than altered cellular phenotypes preferentially drive long‐term loss of muscle mass. Alternatively, the small number of DEGs within each cell type in muscle 1 month post‐infection may be sufficient to lead to long‐lasting defects in muscle post‐sepsis. Future studies investigating the functional role of these DEGs in maintaining muscle mass are warranted. In contrast, adipose tissue exhibited a number of cell populations with sustained transcript alterations, including adipose stem cells and preadipocytes, T‐cells, and macrophages (*Figure* 2D). Considered alongside major shifts in population abundance (*Figure* 2B) and gross changes in tissue mass (*Figure* S9C), these data suggest that compared with skeletal muscle, adipose tissue exhibits less resilience following severe infection. Future studies aimed at understanding the basis of cellular and tissue resilience post‐sepsis would likely accelerate efforts to enhance sepsis survivor outcomes and quality of life.

This study highlights several potential therapeutic avenues to improve post‐sepsis tissue homeostasis: (i) reducing/enhancing the abundance of individual cell populations, (ii) targeting specific signalling pathways to counteract altered gene expression networks, or (iii) targeting classes of molecules (i.e. DAMPs/PAMPs) linked to chronic inflammation/tissue dysfunction. In the first example, pan‐macrophage depletion has been shown to reduce muscle wasting in mouse models of treatment‐associated cachexia,^51^ although caution is certainly warranted with this type of approach given other studies showing positive roles for macrophages in protection from tissue atrophy and muscle regeneration.^52^ Indeed, the optimal strategy might be therapies that act to both boost underrepresented and reduce aberrantly expanded cell populations. An example of this approach could be classes of molecules such as IL‐1β or IL‐17 neutralizing antibodies or NLRP‐3 inflammasome inhibitors that target the M1/M2 macrophage transition, effectively reducing M1‐like macrophage accumulation and enhancing M2‐like macrophage abundance.^53^, ^54^, ^55^ Second, modulation of signalling networks/hubs such as Il‐6 or nitric oxide signalling that are broadly mis‐regulated in multiple cell types (*Figure* 3) may be preferred over indiscriminate population reduction/expansion approaches. Third, as highlighted earlier, therapies that reduce the expression or accumulation of nucleotides, cytokines, or metabolites that collectively comprise the DAMP/PAMP response could potentially limit chronic inflammation/persistent inflammatory catabolism syndrome and improve tissue function. Finally, we conceptually show that molecules identified from upstream regulator analyses of single‐cell data, such as GnRH and Bay 11‐7082, have the potential to counteract tissue loss as a result of severe infection (*Figure* 5C and 5D). Moving forward, we anticipate that single‐cell studies will be increasingly used as discovery engines (i.e. this study) and as validation tools (future work) in order to predict, test, and optimize translational interventions to counteract persistent tissue loss in sepsis survivors.

## Data availability

Datasets in this study are available at Sequence Read Archive under accession number PRJNA626597.

## Author contributions

D. S. C. and J. D. D. designed the study. D. S. C. collected the data. D. S. C. and J. D. D. analysed/interpreted the data. D. S. C., R. E. S., A. D., and A. M. D. performed CLP model derivation/analysis. D. S. C. and J. D. D. wrote and edited the manuscript. All authors approved the final version of the manuscript.

## Conflict of interest

The authors have no competing interests to declare.

## Supporting information

Supporting Information S1

Supporting Information S2
