# Supplementary material for: Single‐cell deconstruction of post‐sepsis skeletal muscle and adipose tissue microenvironments
Source: J Cachexia Sarcopenia Muscle. 2020 Jul 8;11(5):1351–63. doi: 10.1002/jcsm.12596 (PMC7567136; doi:10.1002/jcsm.12596)
Supplement: Supplementary file 2 — Supporting Information S2 [file JCSM-11-1351-s002.pdf]

SUPPLEMENTARY FIGURES

Single cell deconstruction of post-sepsis skeletal muscle and adipose tissue microenvironments

Dong Seong Cho[1], Rebecca E Schmitt[1], Aneesha Dasgupta[1], Alexandra M Ducharme[1], Jason D Doles[1]*

[1]Department of Biochemistry and Molecular Biology, Mayo Clinic, Rochester, Minnesota, 55905 USA.

*Corresponding Author:
Jason D Doles
Department of Biochemistry and Molecular Biology
Mayo Clinic
200 First St SW
Guggenheim 16-11A1
Rochester, MN 55905
Tel: (507) 284-9372
Fax: (507) 284-3383
E-mail: Doles.Jason@mayo.edu

Supplementary Figure S1.

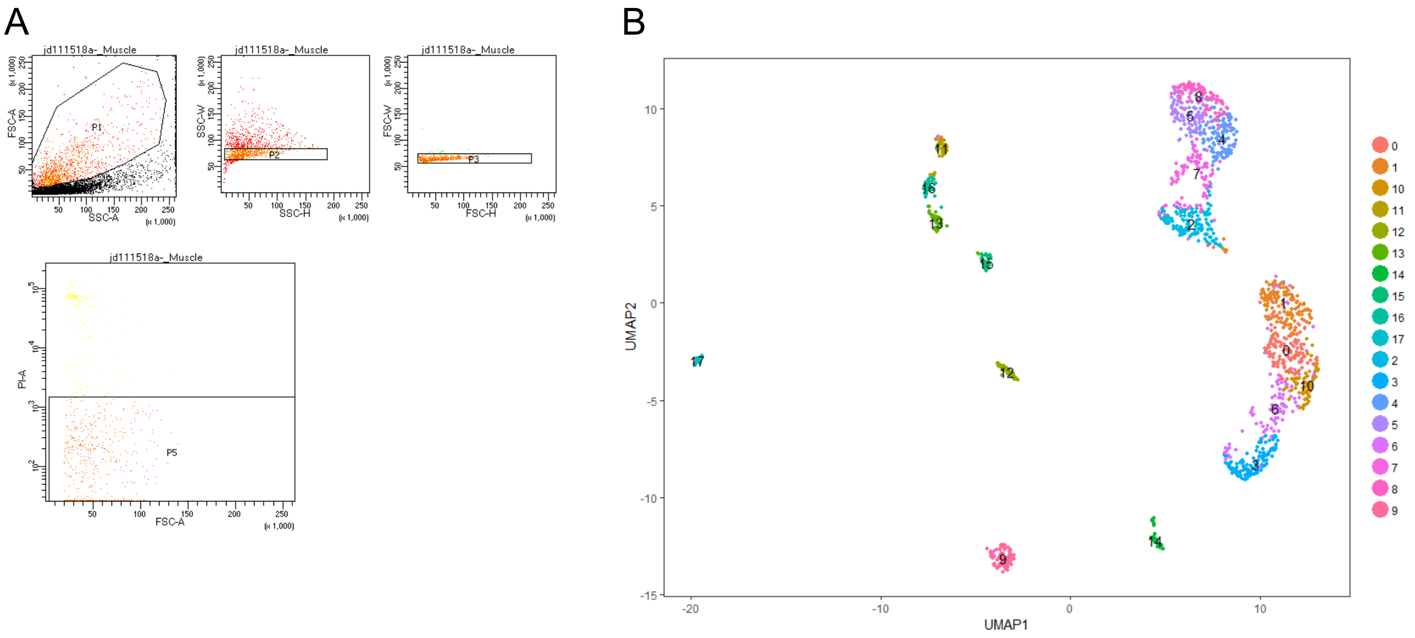

Supplementary Figure S1: Single-cell RNA-sequencing (scRNA-seq) analysis on mouse muscle tissues. (A) Gating strategy to isolate propidium iodide^{neg} single cells from mouse tibialis anterior (TA) muscle by fluorescence activated cell sorting (FACS). (B) Uniform manifold approximation and projection (UMAP) of muscle single cells clustered into 18 clusters.

Supplementary Figure S2.

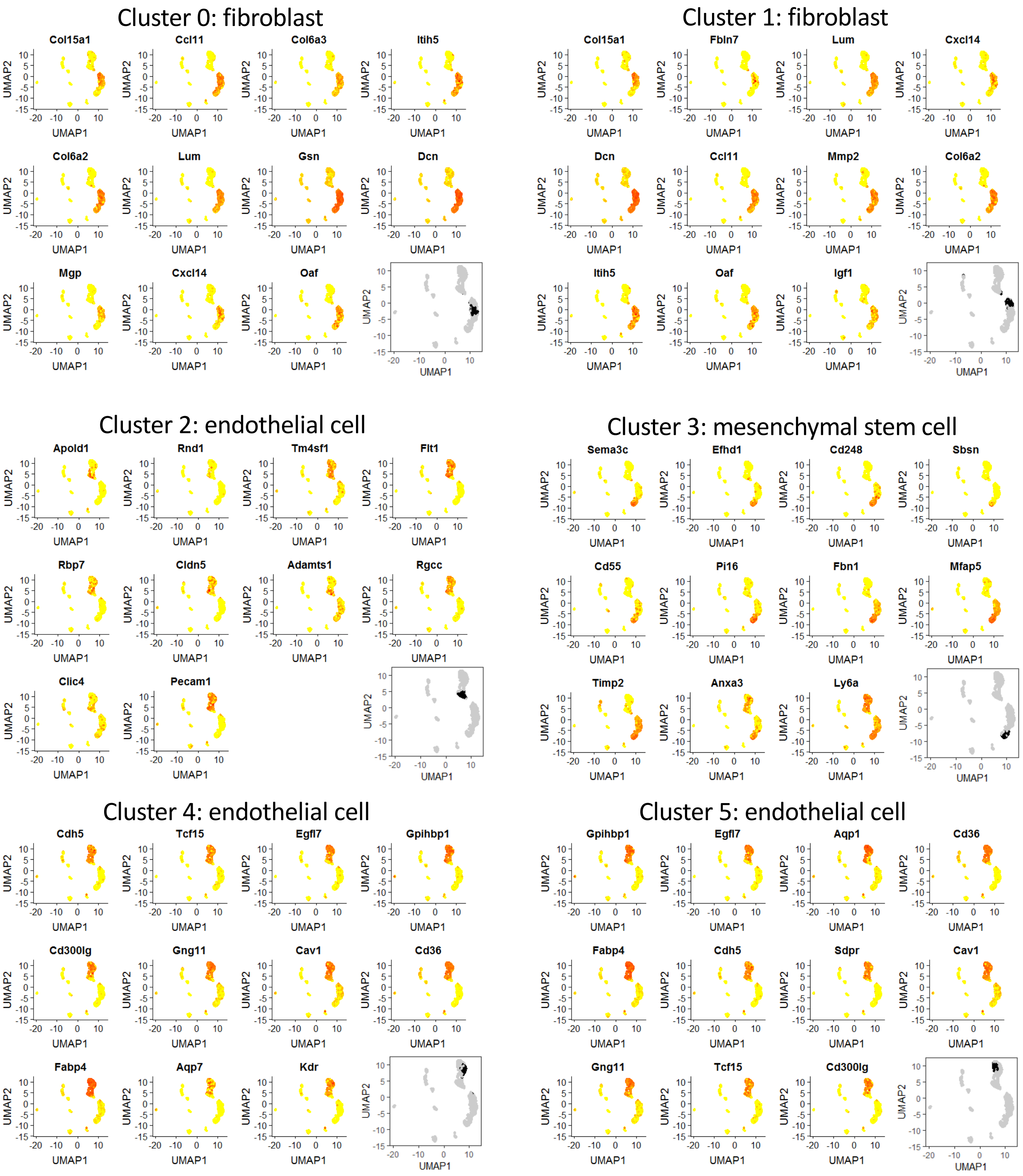

Supplementary Figure S2. (cont)

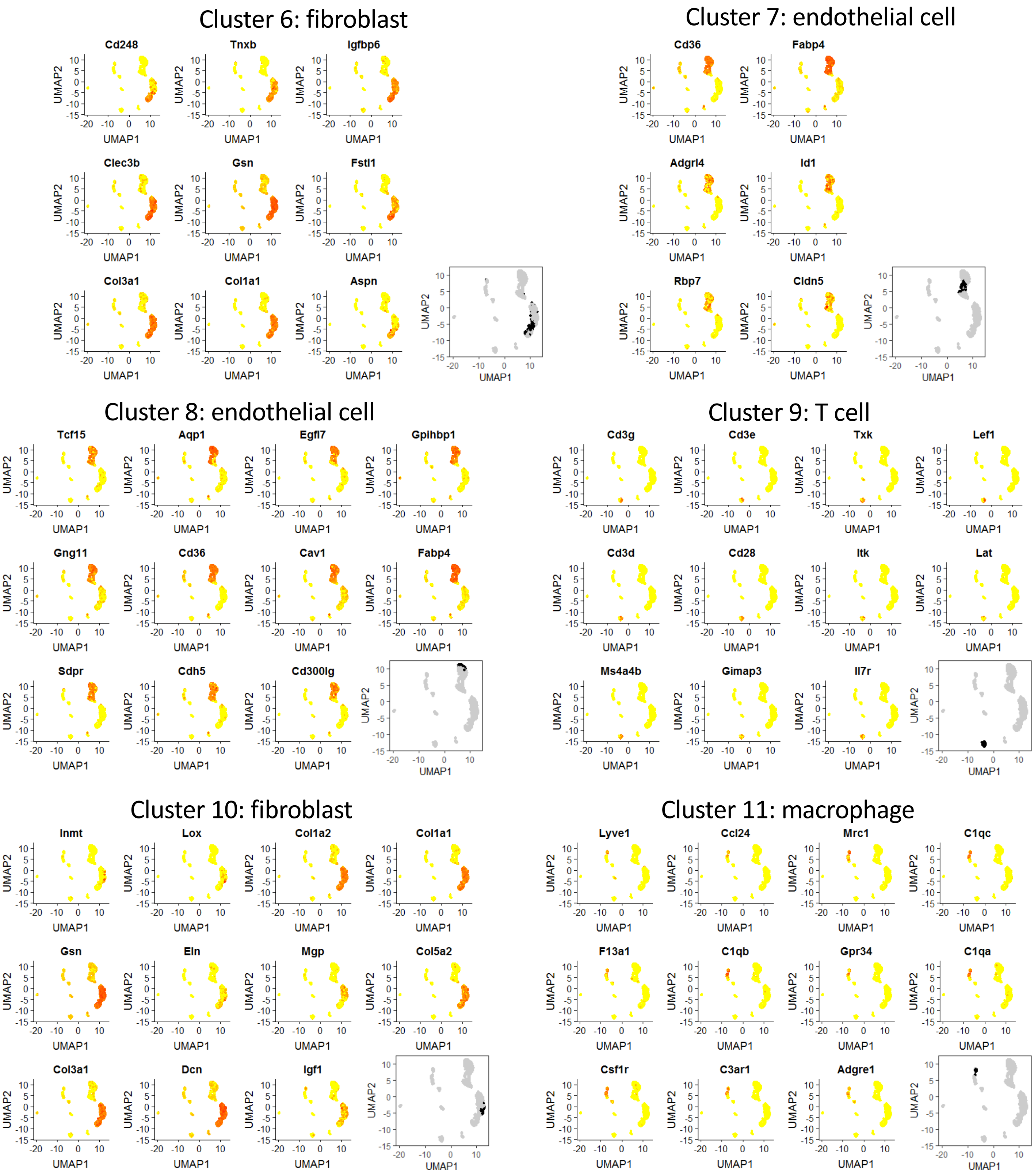

Supplementary Figure S2. (cont)

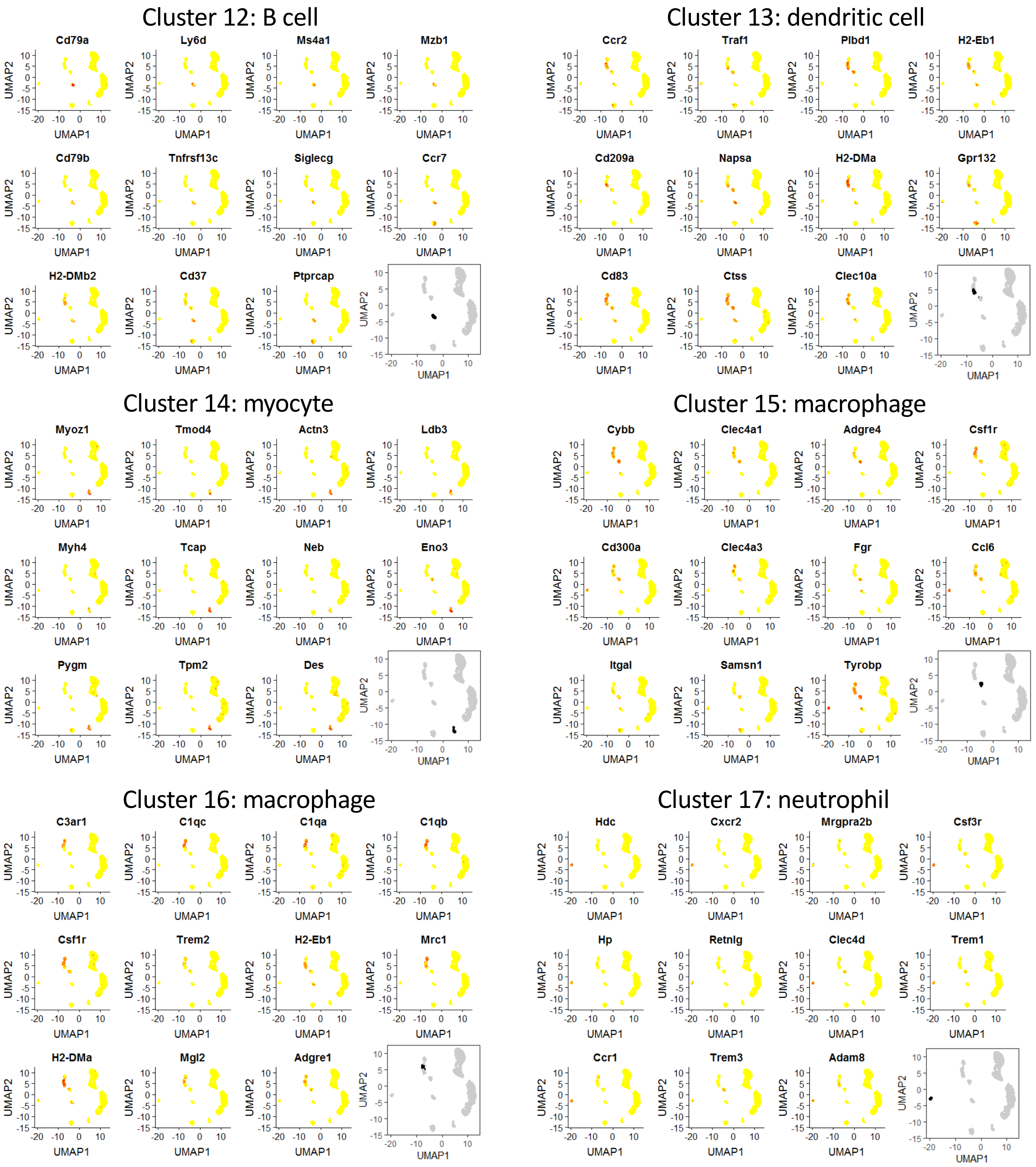

Supplementary Figure S2. (cont)

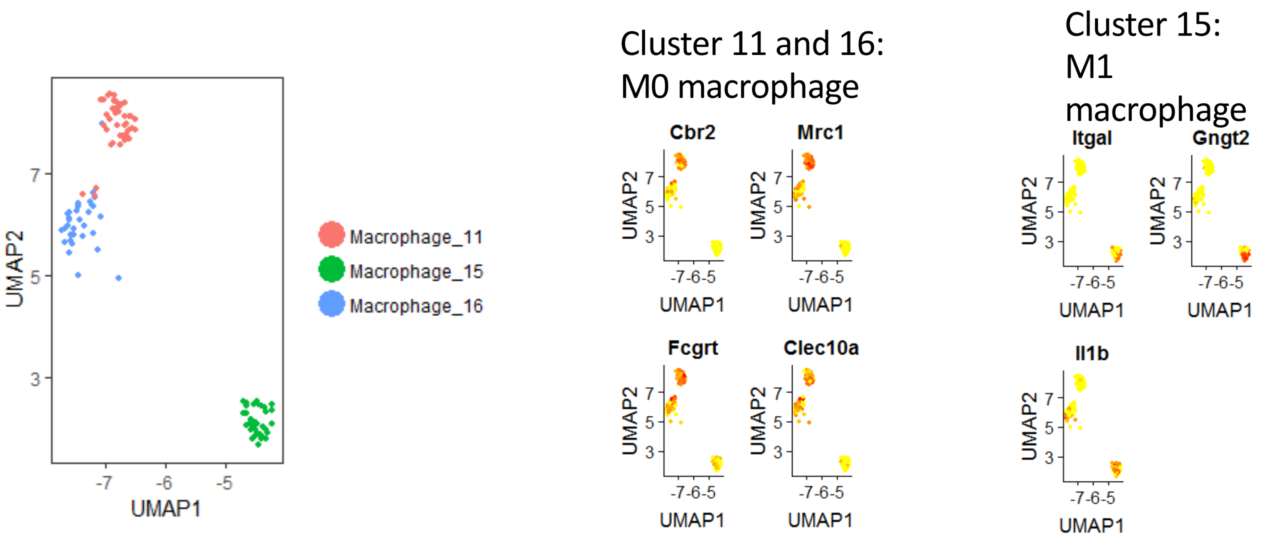

Supplementary Figure S2. Classification of 18 clusters of muscle cells into 10 cell types based on differentially expressed genes (DEGs). Expression of cell type markers that are differentially expressed in each cluster is shown. Colors in UMAP projections represent expression levels of genes with yellow=low/not expressed and red/orange=highly expressed. UMAP projection in the right bottom corner highlights the cells of a given cluster. After clusters 11, 15, and 16 were classified into macrophages, they were sub-classified into M0 and M1 macrophages based on DEGs between these clusters.

Supplementary Figure S3.

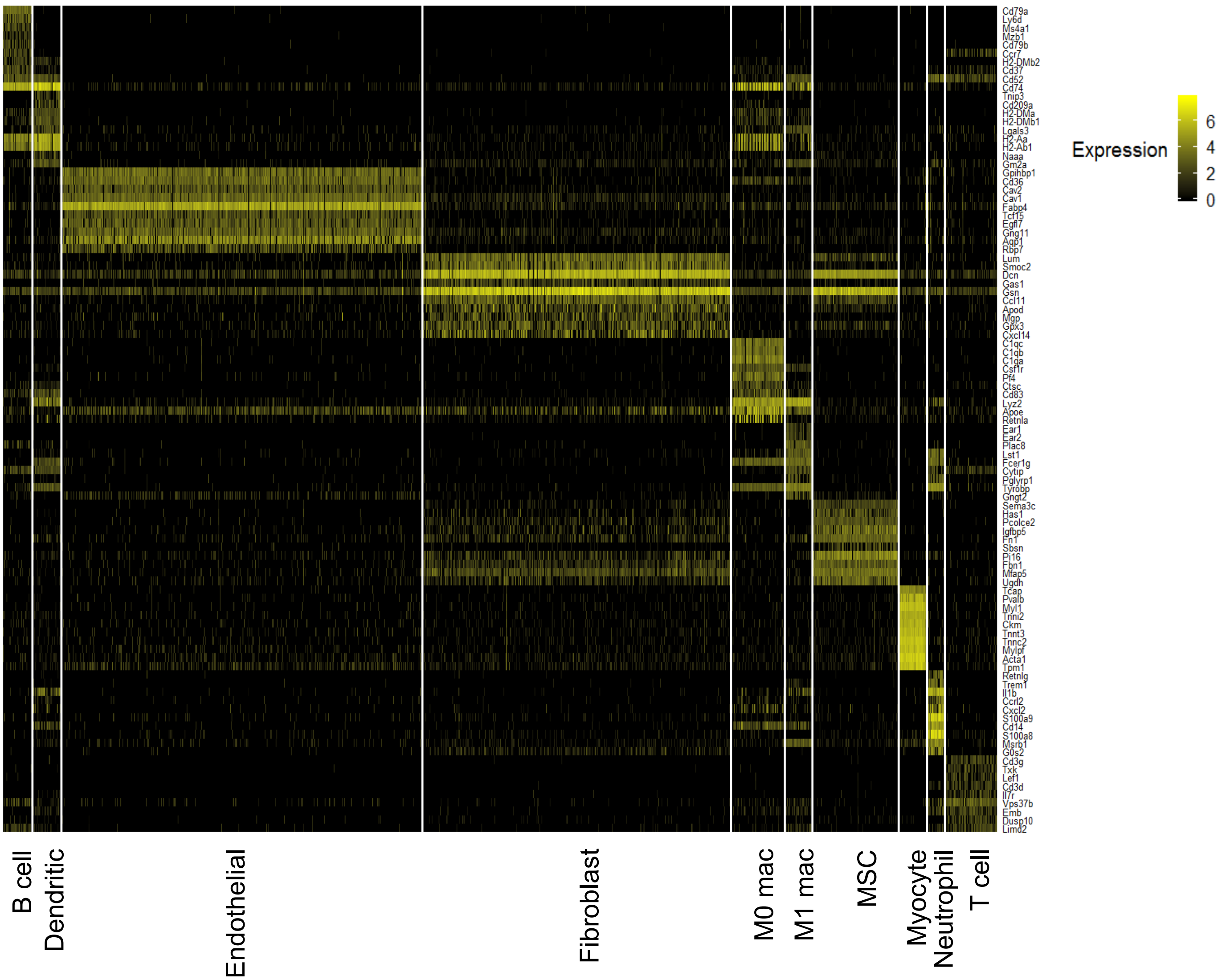

Supplementary Figure S3: Top 10 markers of each cell type in mouse muscle tissues. A heatmap depicting the top 10 markers (differentially expressed genes) of each main cell type. Yellow=high relative expression, black=low relative expression.

Supplementary Figure S4.

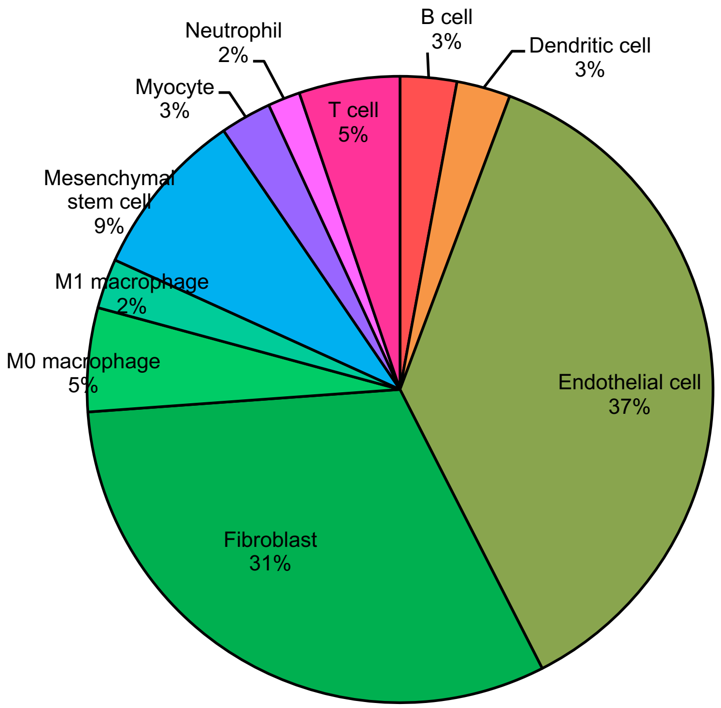

Supplementary Figure S4: A pie chart depicting the relative proportion of each cell type in the TA muscle microenvironment.

Supplementary Figure S5.

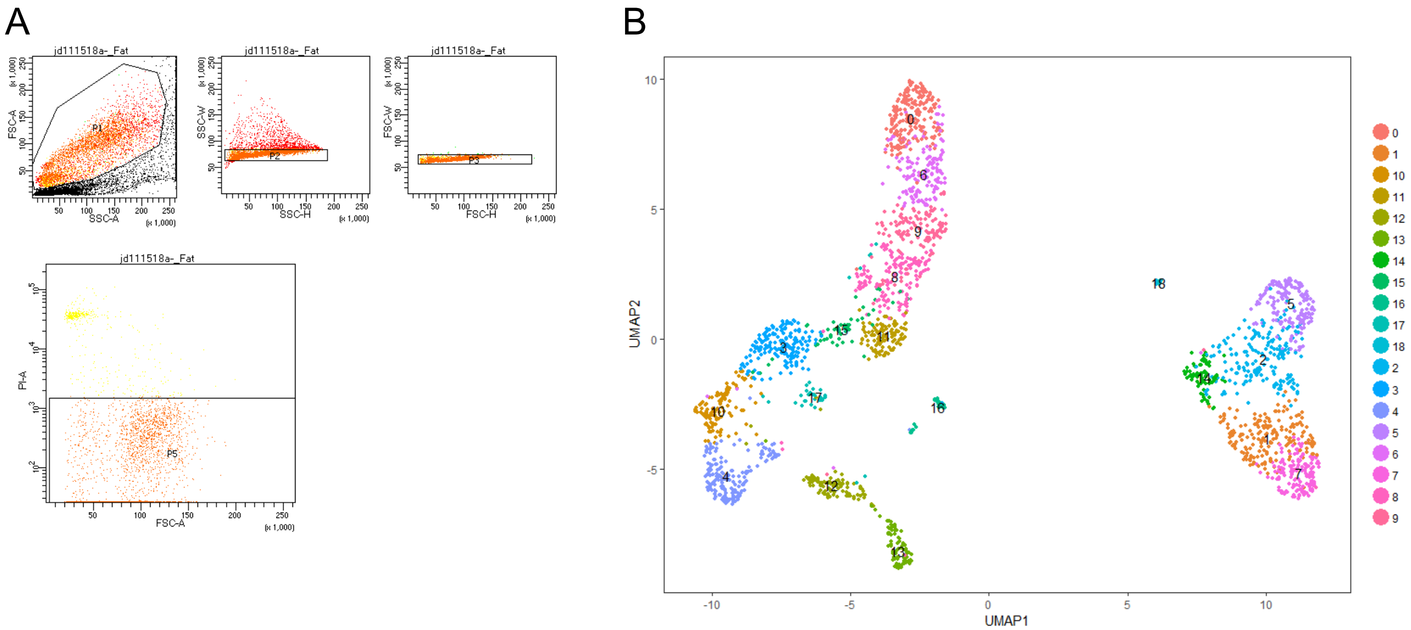

Supplementary Figure S5: ScRNA-seq analysis on mouse visceral adipose tissue. (A) Gating strategy to isolate propidium iodide^{neg} single cells from mouse epididymal fat pads by fluorescence activated cell sorting (FACS). (B) Uniform manifold approximation and projection (UMAP) of muscle single cells clustered into 19 clusters.

Supplementary Figure S6.

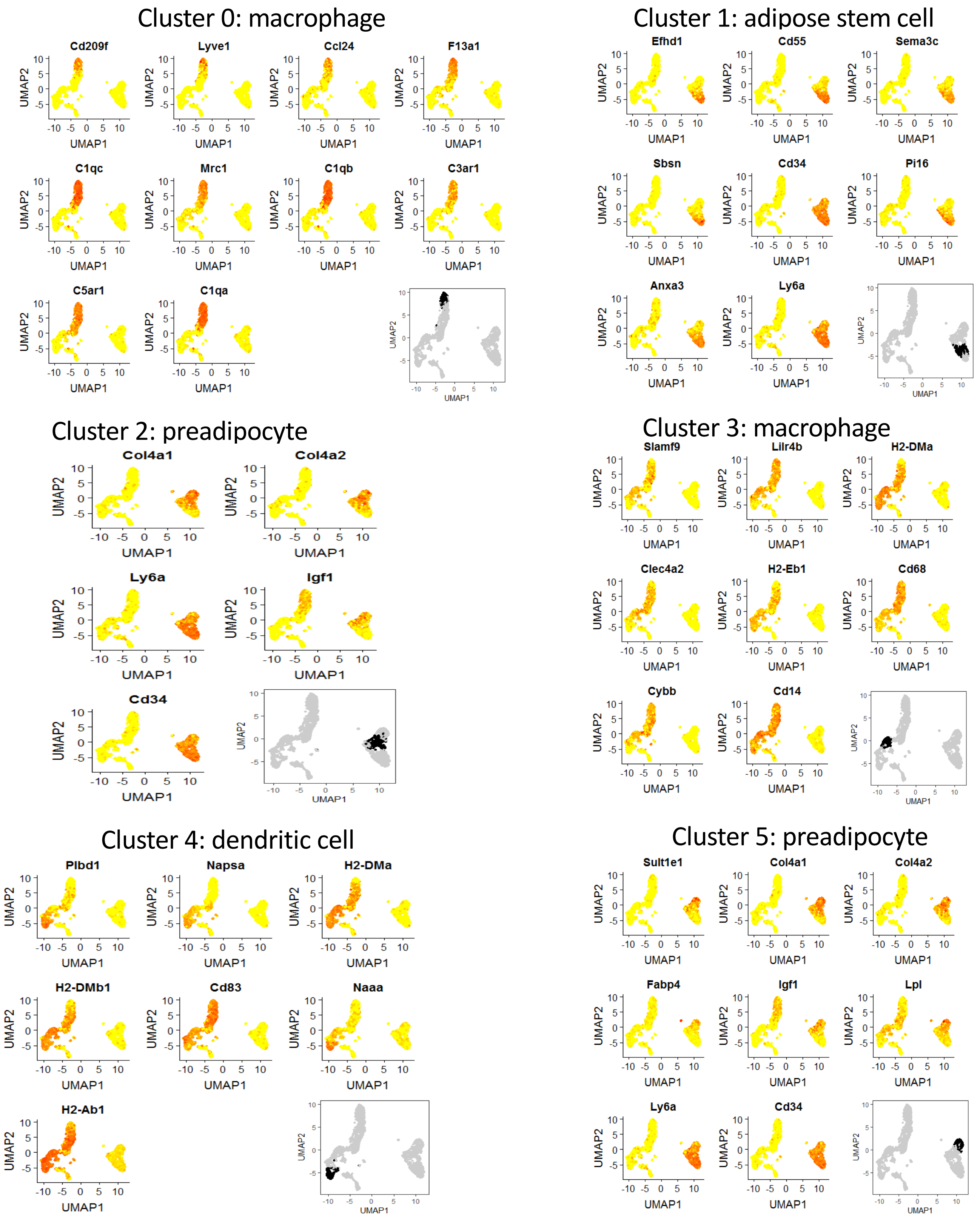

Supplementary Figure S6. (cont)

Cluster 6: macrophage

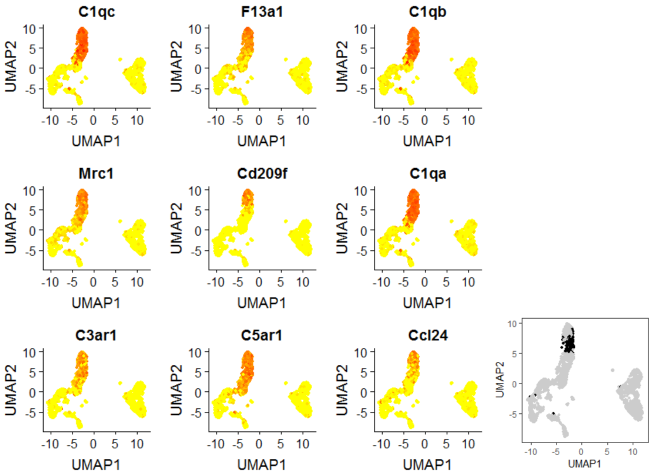

Cluster 7: adipose stem cell

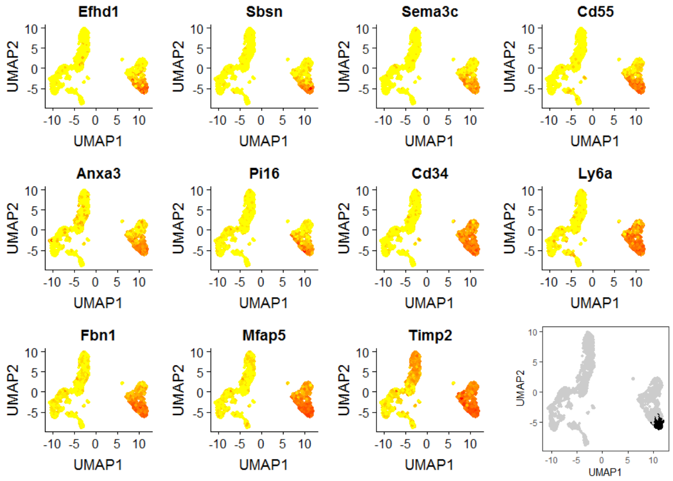

Cluster 8: macrophage

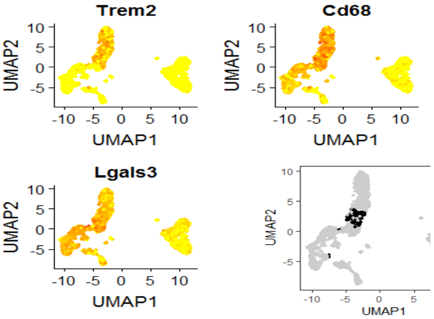

Cluster 9: macrophage

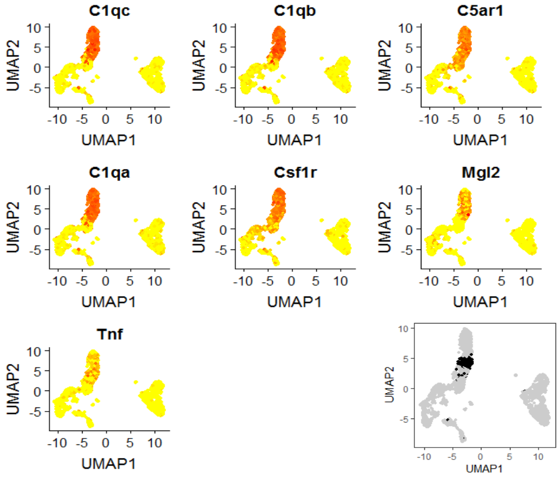

Cluster 12: T cell

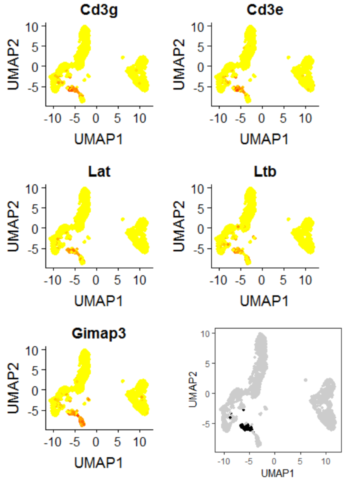

Cluster 10: dendritic cell

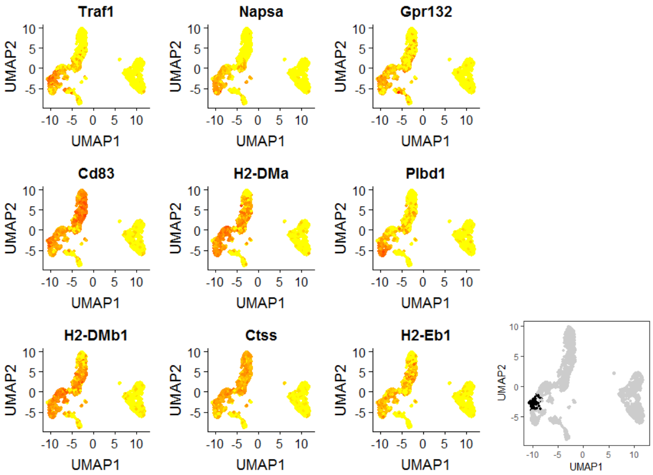

Cluster 11: macrophage

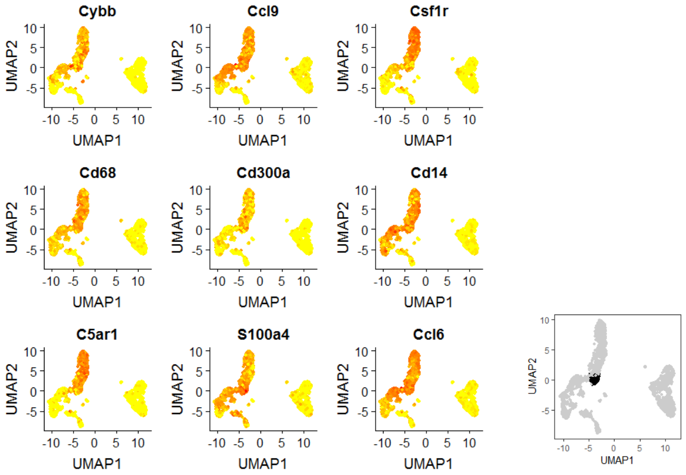

Supplementary Figure S6. (cont)

Cluster 13: NK cell

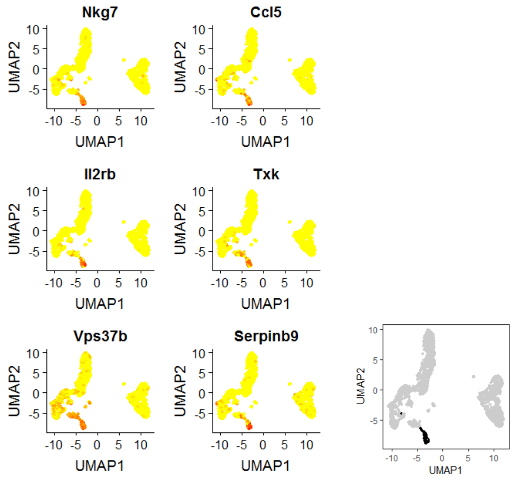

Cluster 14: fibroblast

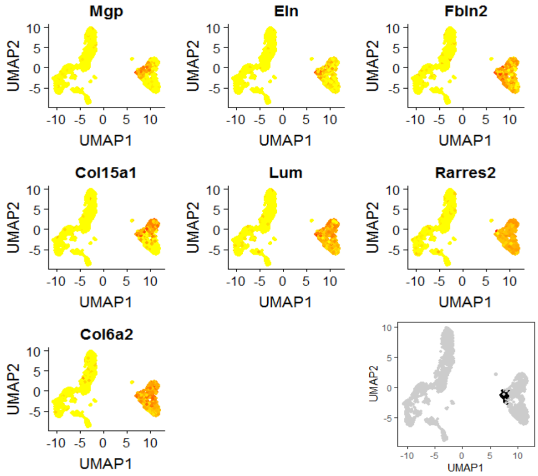

Cluster 15: macrophage

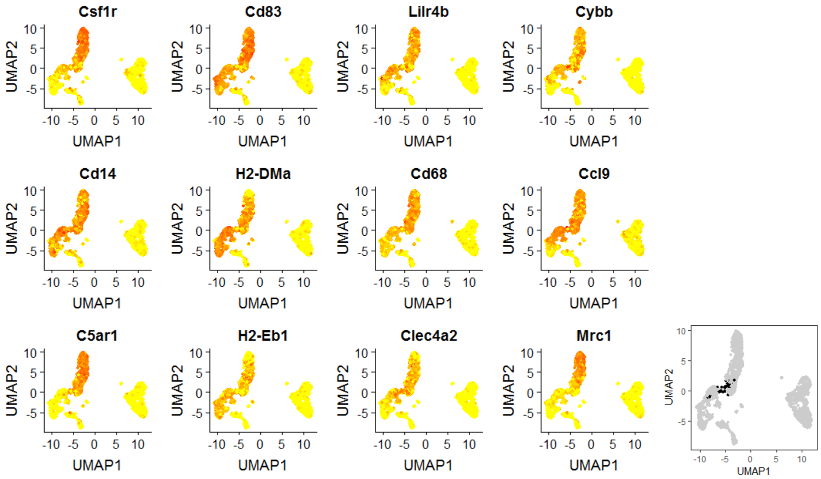

Cluster 16: B cell

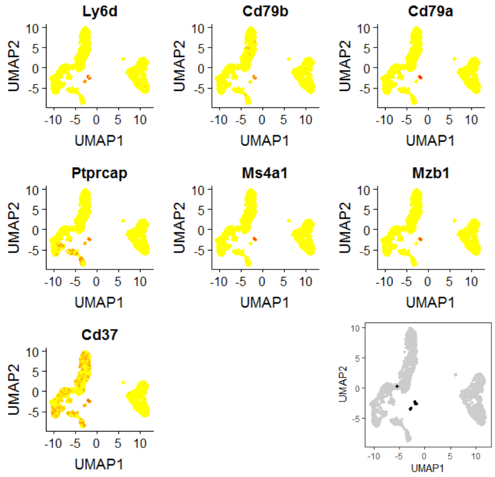

Cluster 17: gamma delta T cell

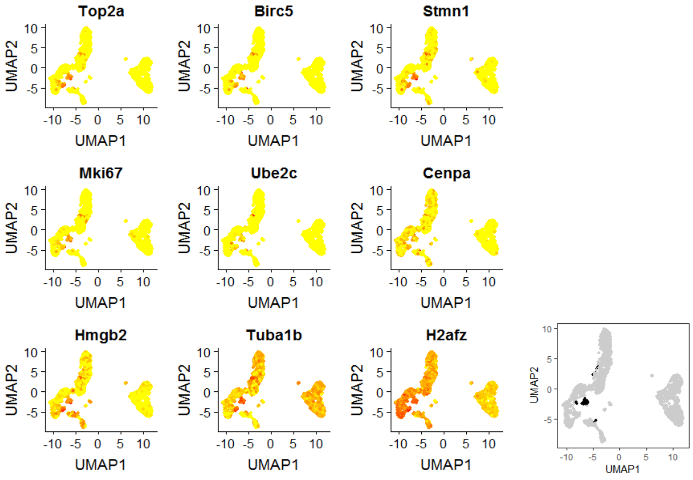

Cluster 18: endothelial cell

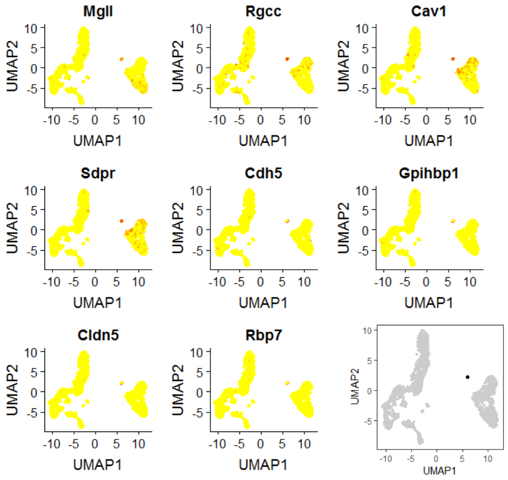

Supplementary Figure S6. (cont)

Supplementary Figure S6: Classification of 19 clusters of adipose tissue cells into 10 cell types based on differentially expressed genes (DEGs). As in Figure S2, expression of cell type markers that are differentially expressed in each cluster are shown with colors (yellow=low/not expressed and red/orange=highly expressed) denoting relative transcript expression levels.

Supplementary Figure S7.

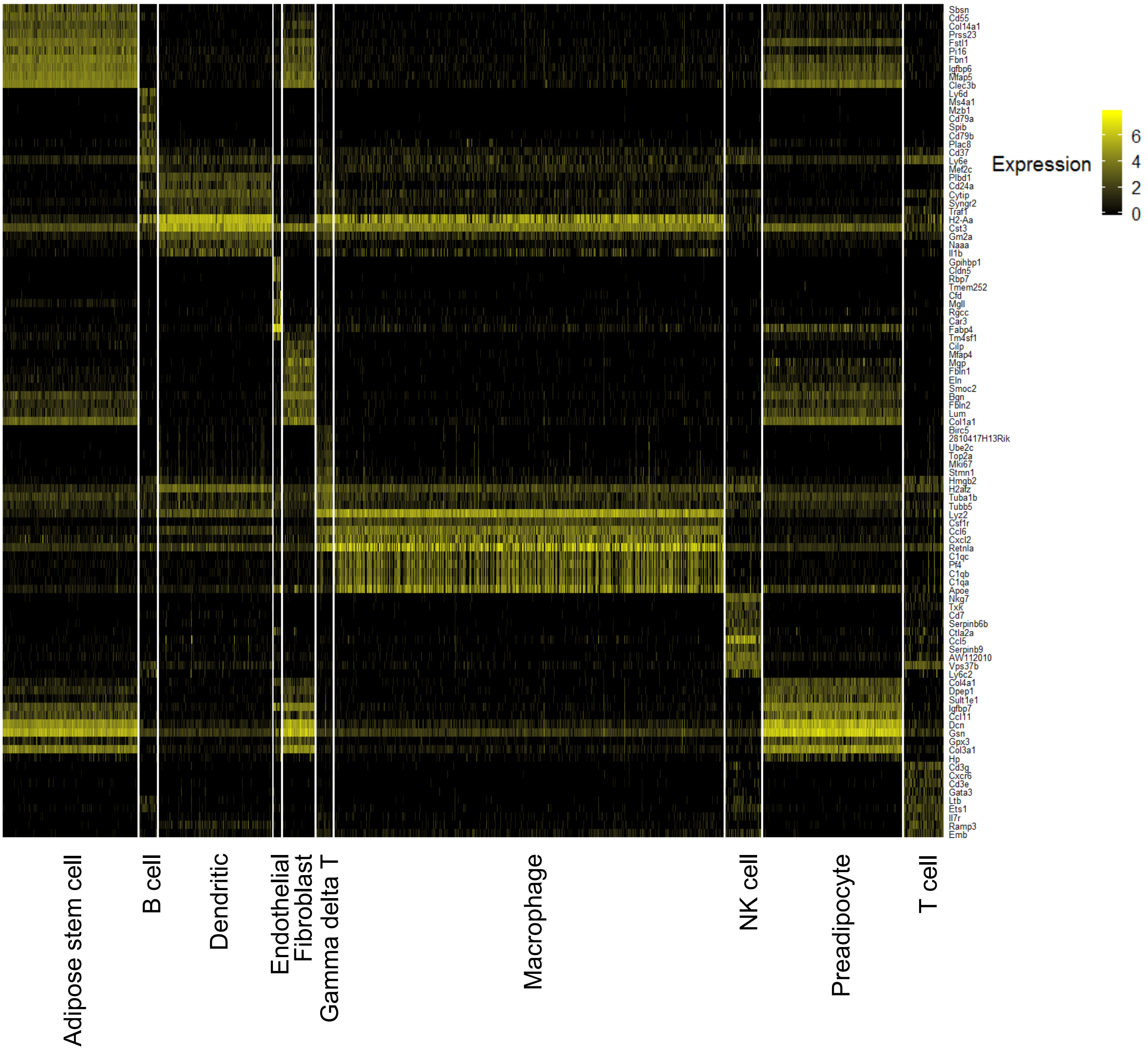

Supplementary Figure S7: Top 10 markers of each cell type in mouse adipose tissues. A heatmap depicting the top 10 markers (differentially expressed genes) of each main cell type. Yellow=high relative expression, black=low relative expression.

Supplementary Figure S8.

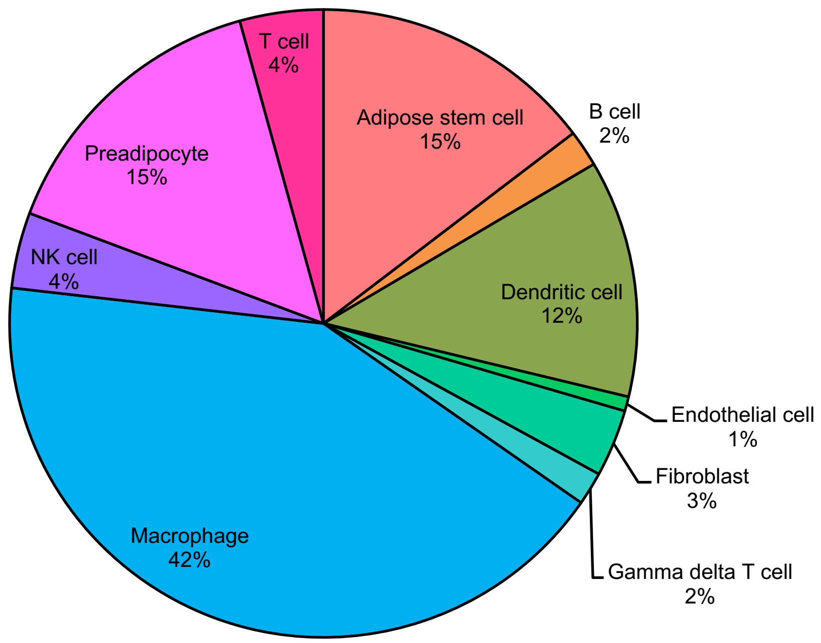

Supplementary Figure S8: A pie chart depicting the relative proportion of each cell type in the adipose tissue microenvironment.

Supplementary Figure S9.

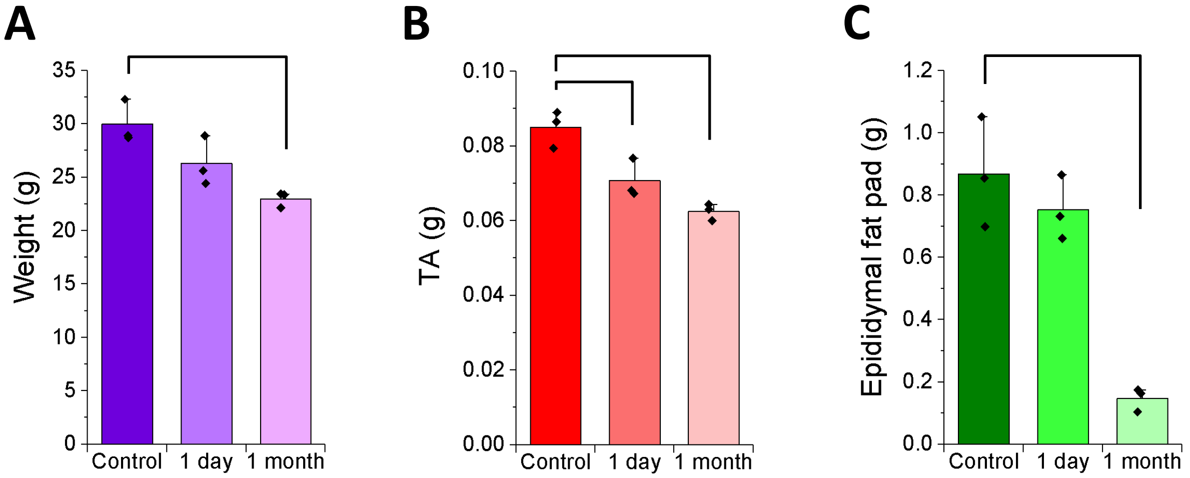

Supplementary Figure S9: Fecal slurry (FS)-induced sepsis (FIP) remodels the muscle and adipose single cell landscape. (A-C) Bar graphs depicting total body weights (A), TA muscle wet weights (B), and epididymal fat pad wet weights (C). Marked comparison denotes statistically significant difference (p-value < 0.05, Student's t-test). N=3 mice per experimental group.

Supplementary Figure S10.

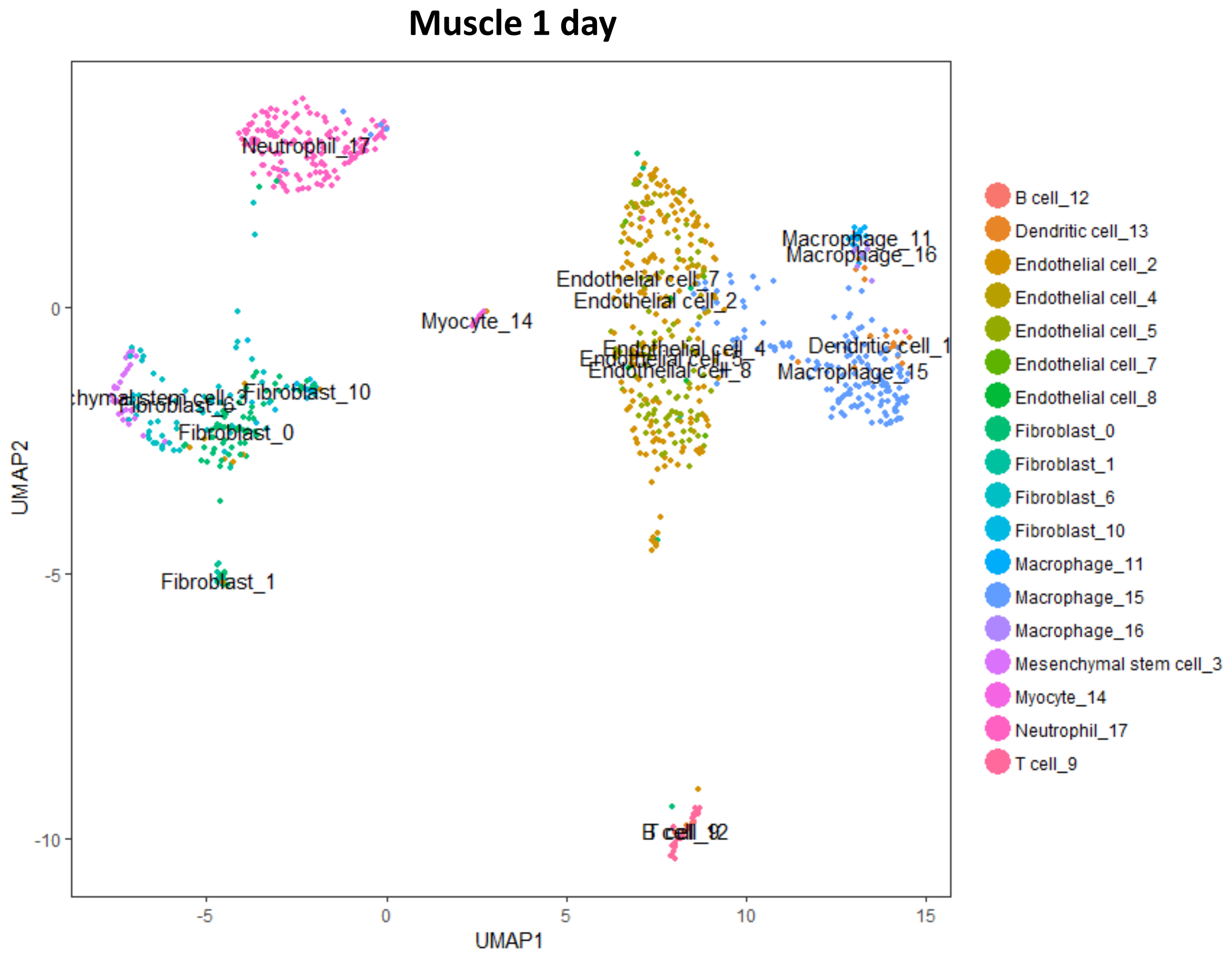

Cluster 0: fibroblast

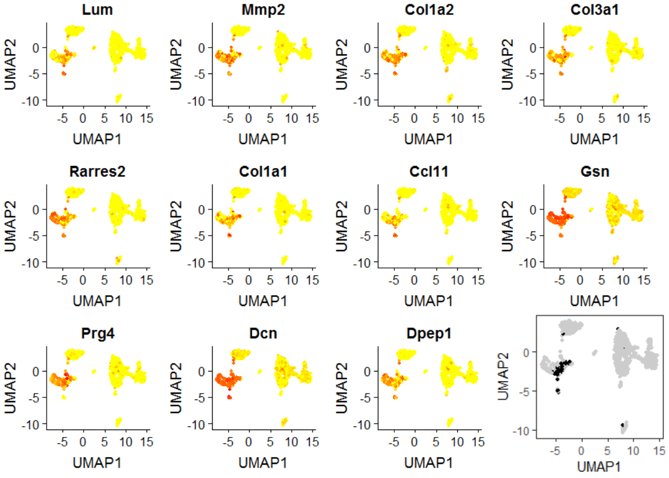

Cluster 1: fibroblast

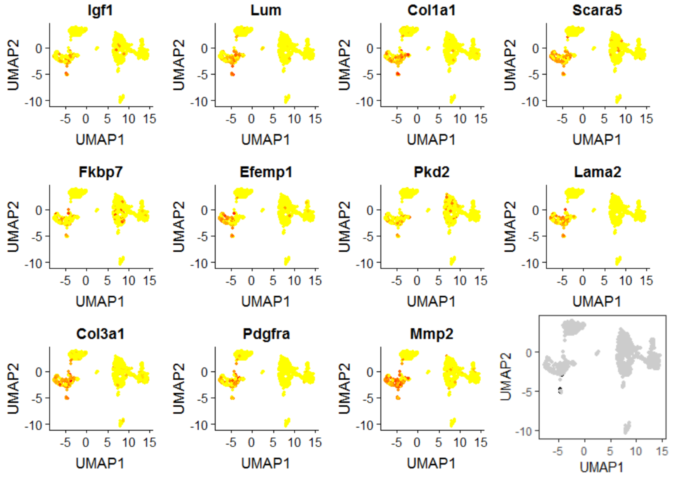

Supplementary Figure S10. (cont)

Cluster 2: endothelial cell

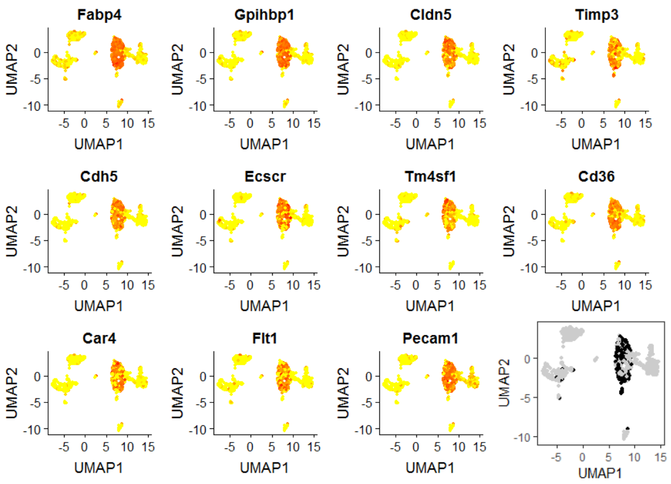

Cluster 3: mesenchymal stem cell

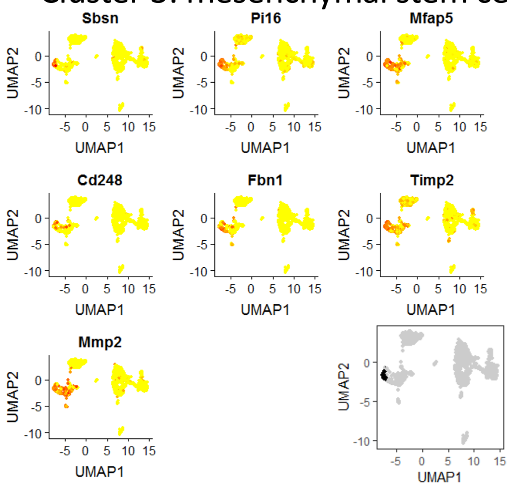

Cluster 4: endothelial cell

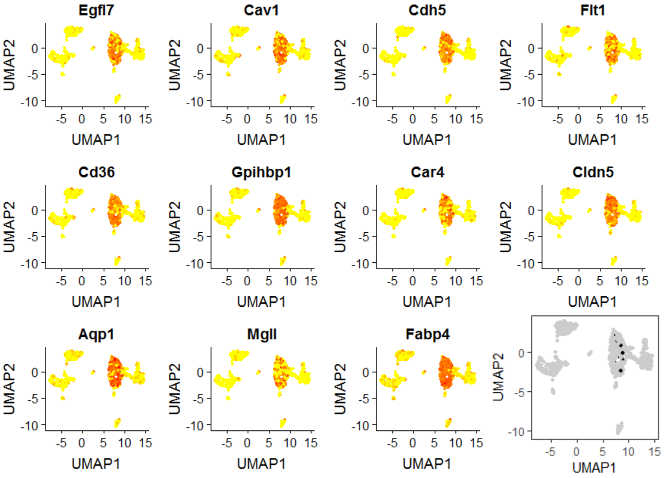

Cluster 5: endothelial cell

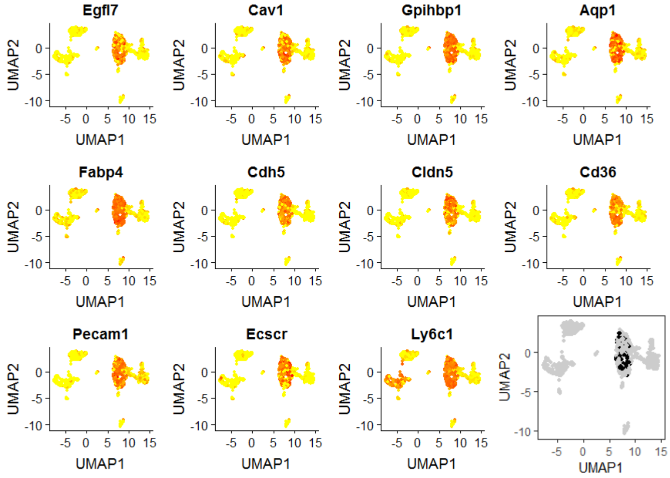

Cluster 6: fibroblast

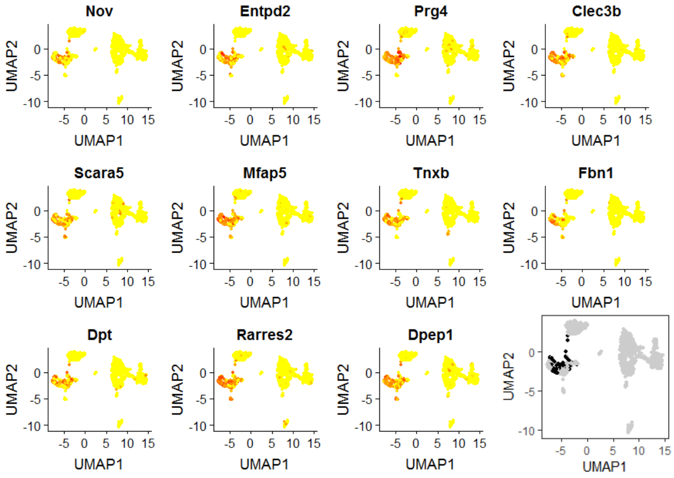

Cluster 7: endothelial cell

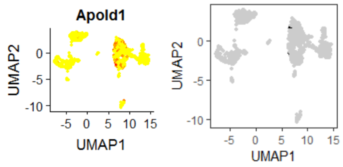

Cluster 8: endothelial cell

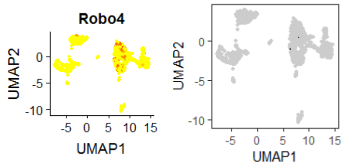

Supplementary Figure S10. (cont)

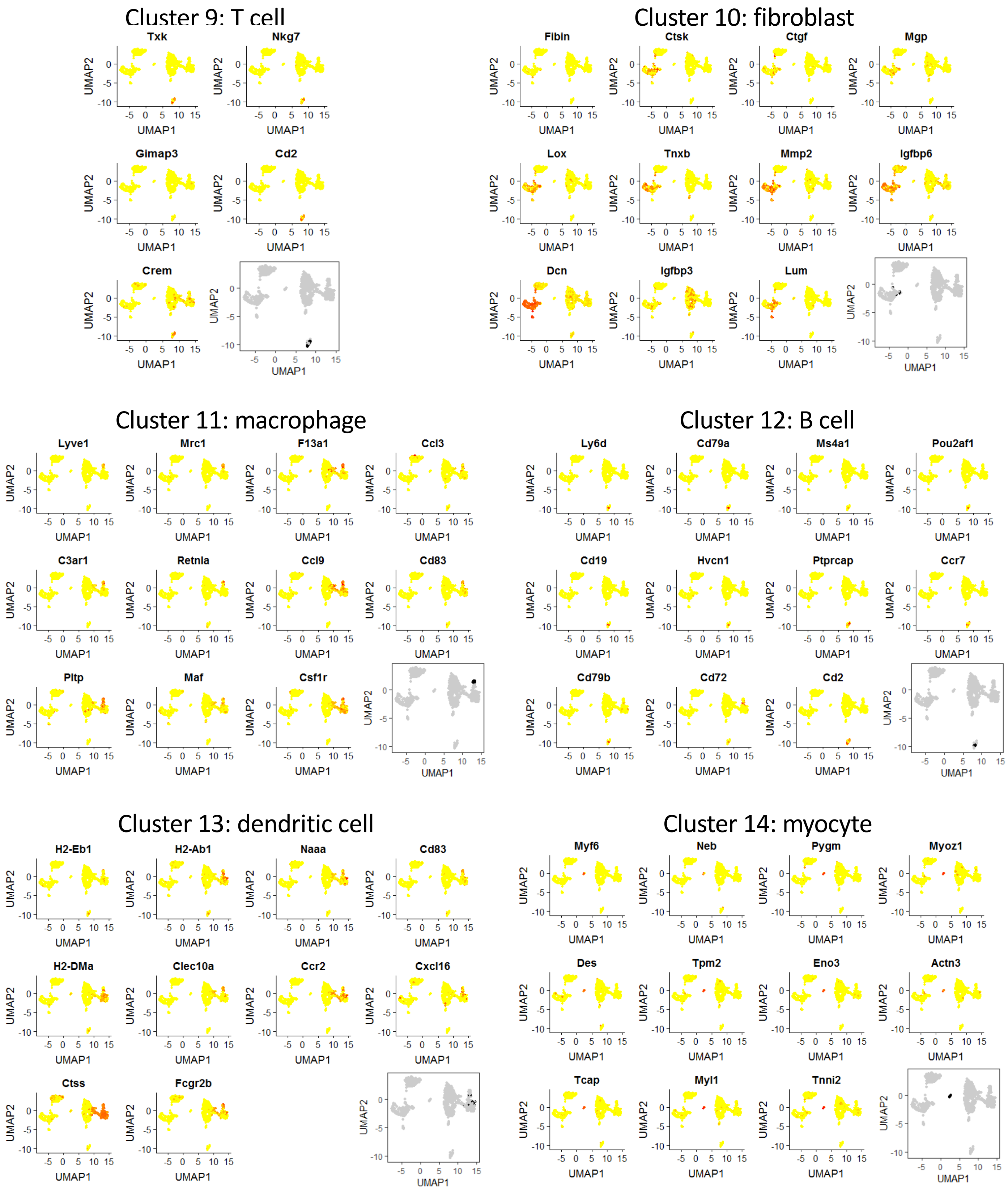

Supplementary Figure S10. (cont)

Cluster 15: macrophage

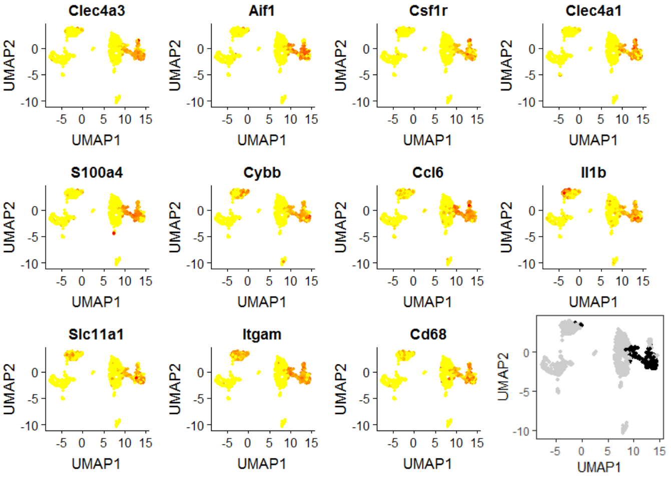

Cluster 16: macrophage

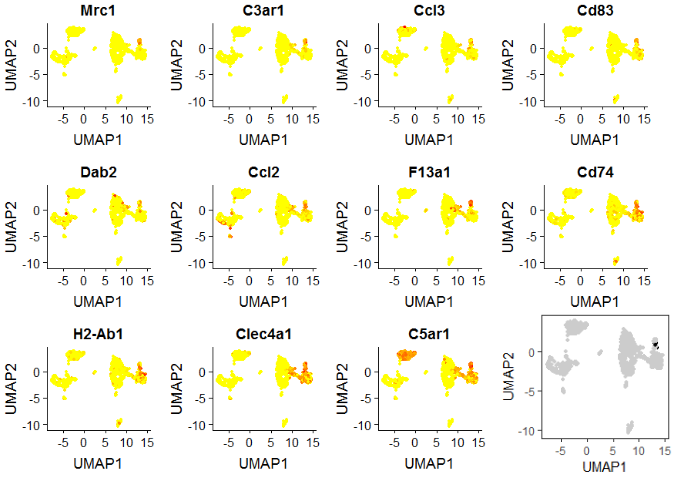

Cluster 17: neutrophil

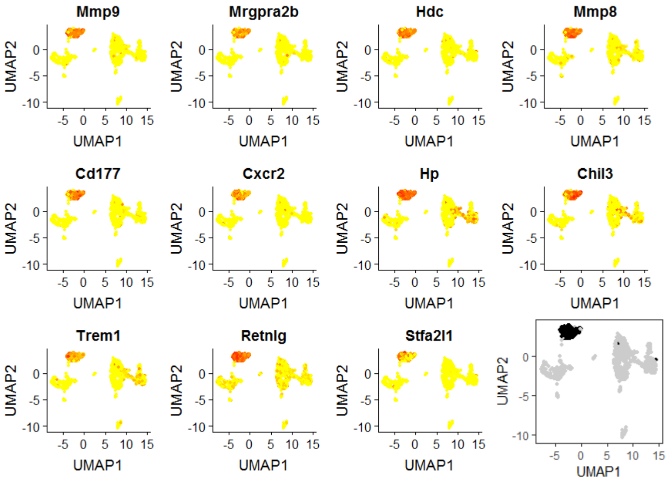

Supplementary Figure S10. (cont)

Macrophages in muscle 1 day

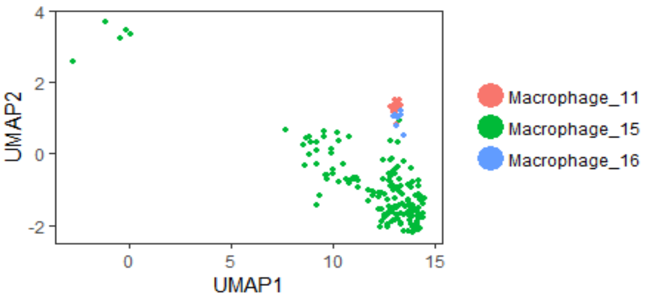

Macrophage 15 subsets

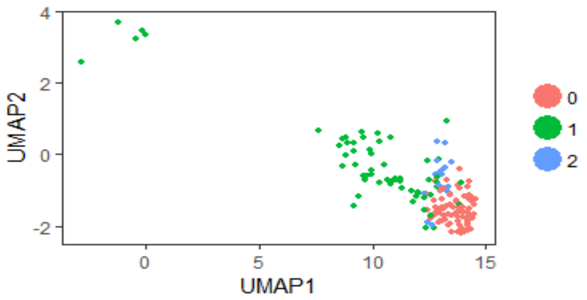

Cluster 11 and 16: M0 macrophage

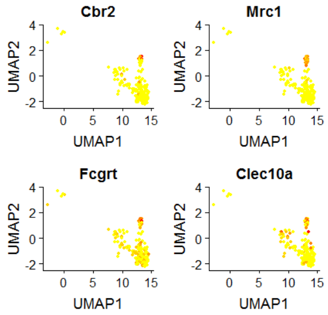

Cluster 15_0 and 15_2: M1 macrophage

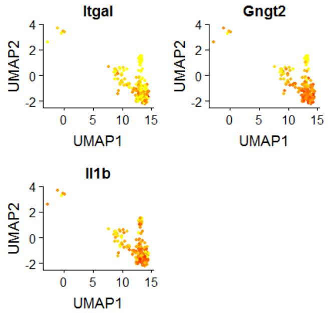

Cluster 15_1: M2 macrophage

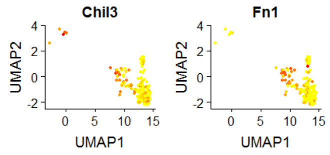

Supplementary Figure S10: UMAP projection of muscle cells 1 day post sepsis. Cell type classifications of muscle cells in control tissue was used to train and classify the cells 1 day post sepsis. Cell type markers that are differentially expressed are shown for each cluster to assess and confirm cell type classification. Comparison between macrophage populations (clusters 11, 15, and 16) re-classified clusters 11 and 16 into M0 macrophages. Cluster 15 were sub-clustered and further classified into M1 and M2 macrophages.

Supplementary Figure S11.

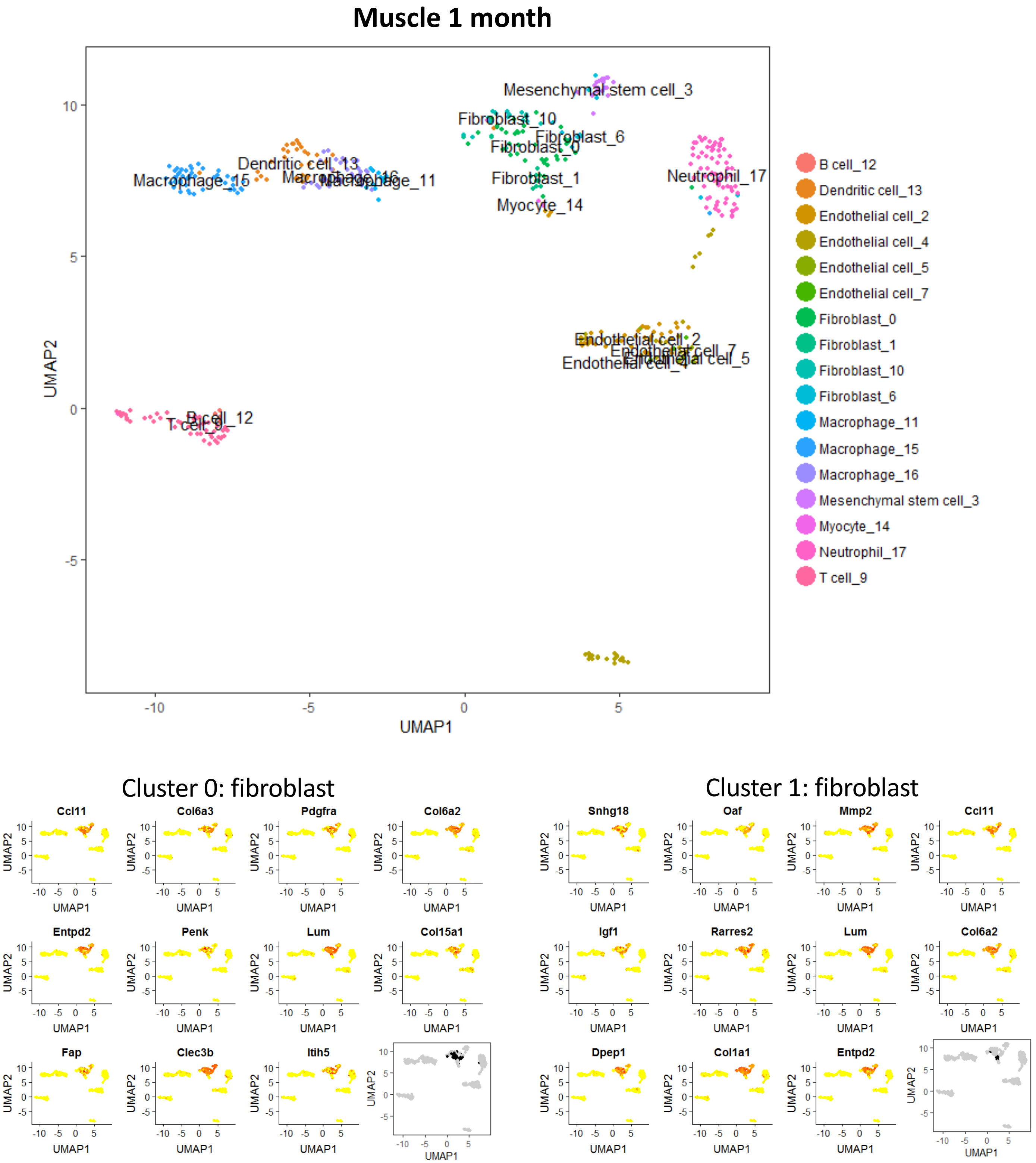

Supplementary Figure S11. (cont)

Cluster 2: endothelial cell

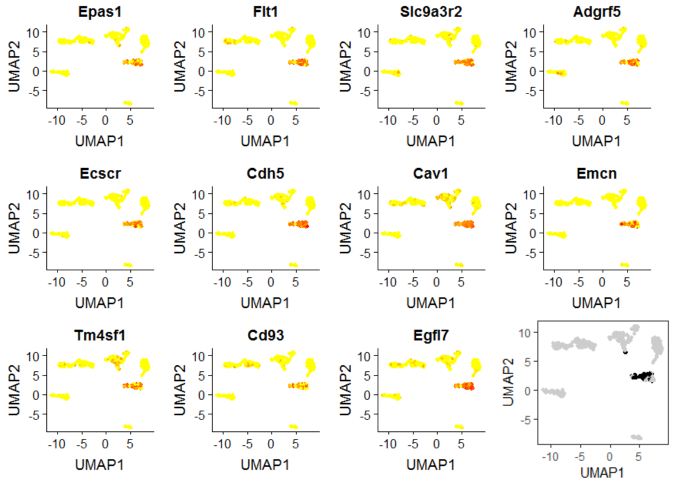

Cluster 3: mesenchymal stem cell

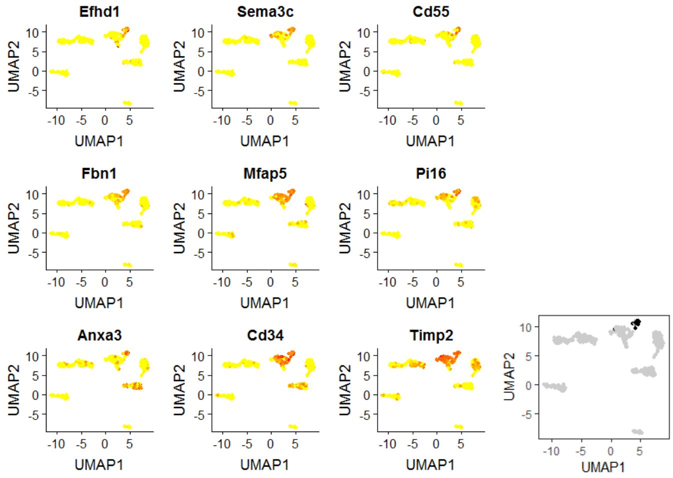

Endothelial cell_4 in muscle 1 month

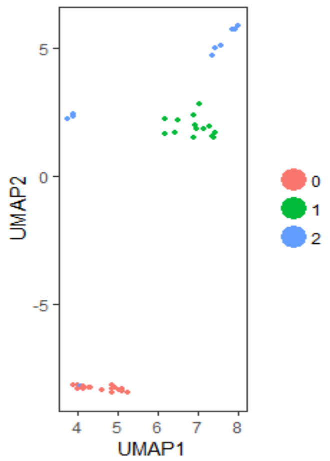

Cluster 4_0: erythroid cell

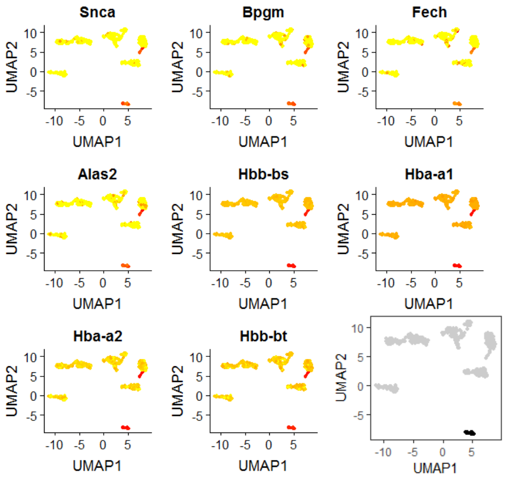

Cluster 4_1: endothelial cell

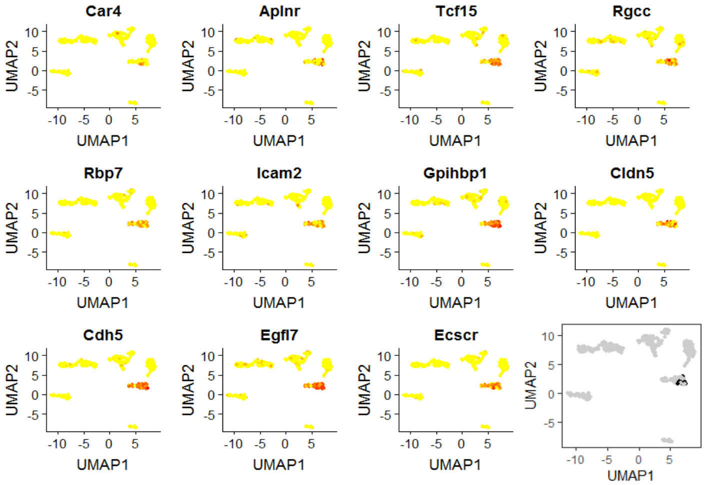

Cluster 4_2: erythroid cell

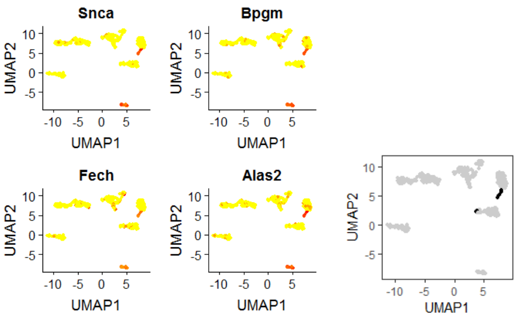

Supplementary Figure S11. (cont)

Cluster 5: endothelial cell

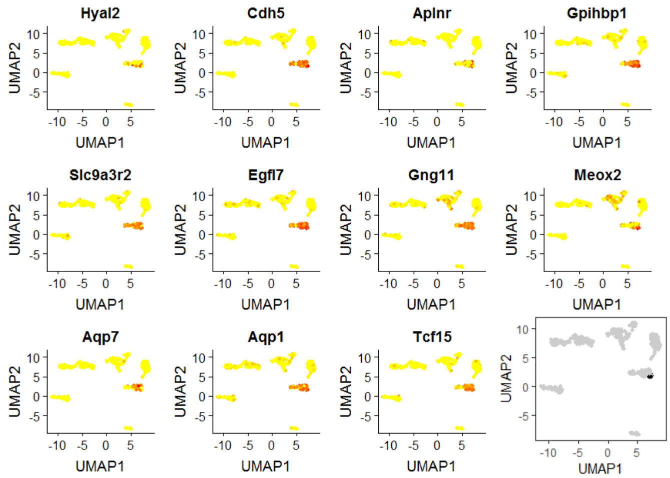

Cluster 7: endothelial cell

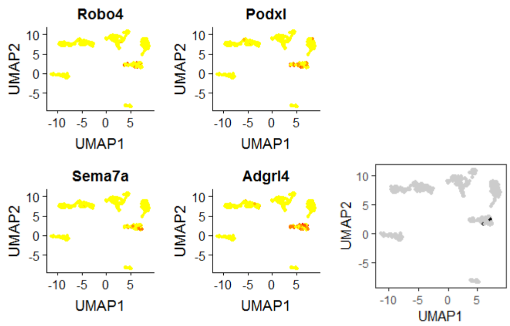

Fibroblast_6 in muscle 1 month

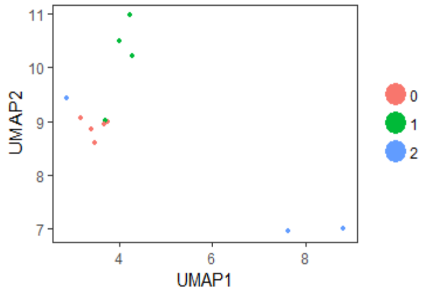

Cluster 6_0: fibroblast

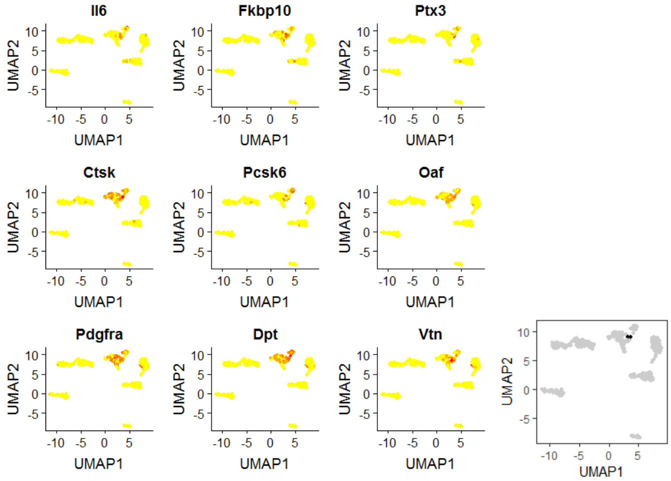

Cluster 6_1: mesenchymal stem cell

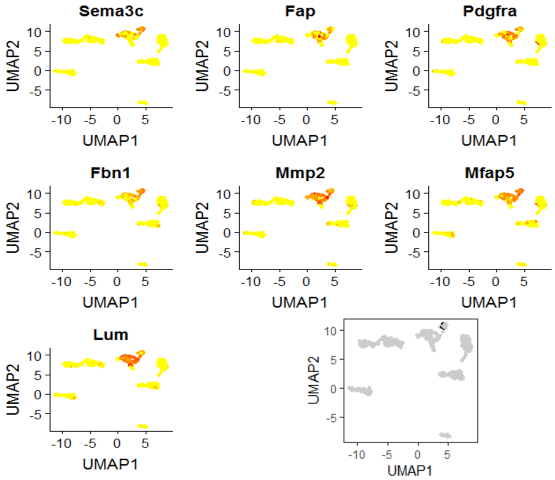

Cluster 6_2: fibroblast

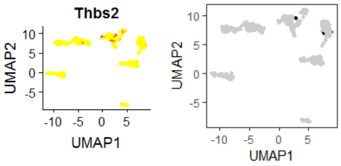

Supplementary Figure S11. (cont)

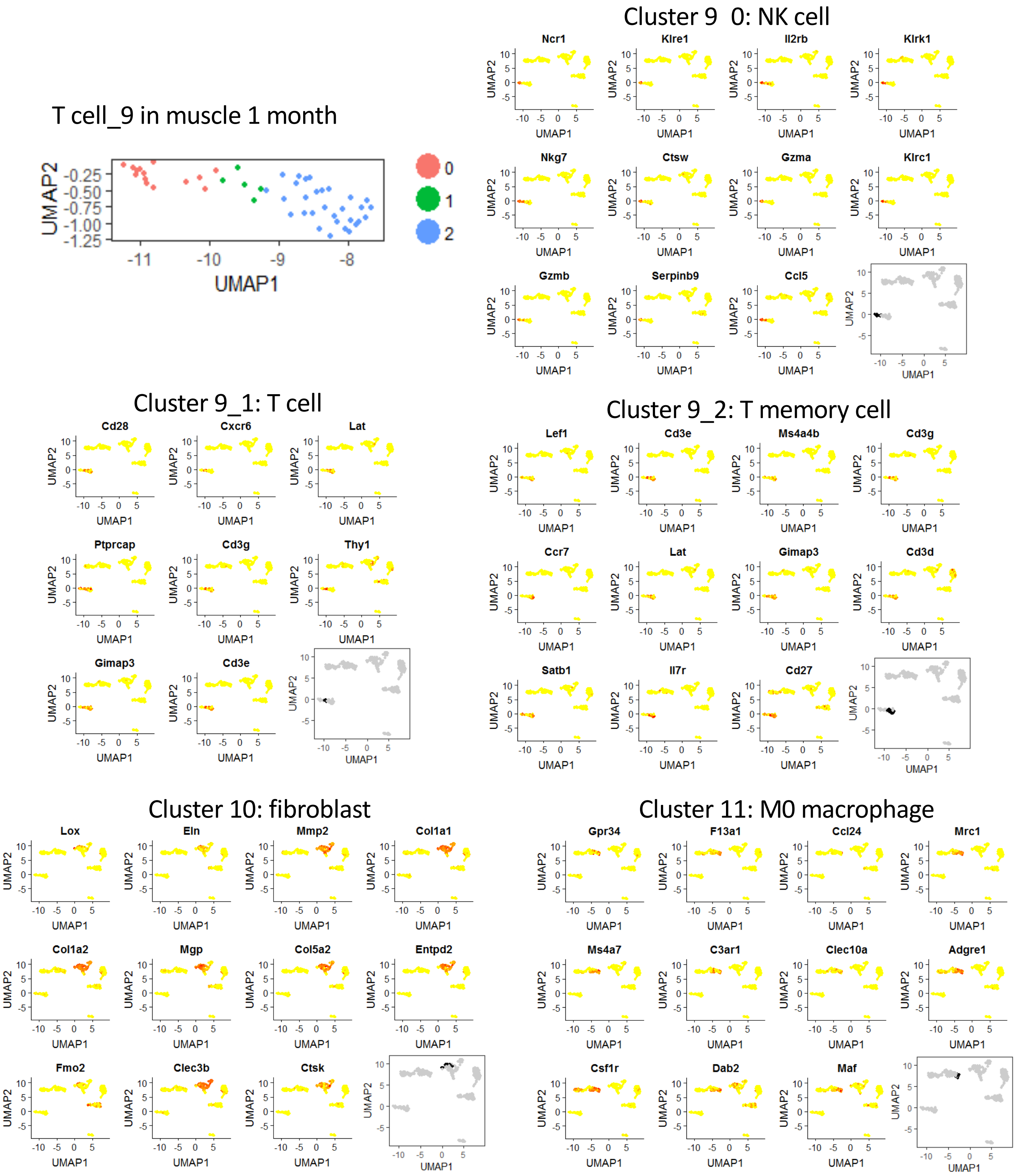

Supplementary Figure S11. (cont)

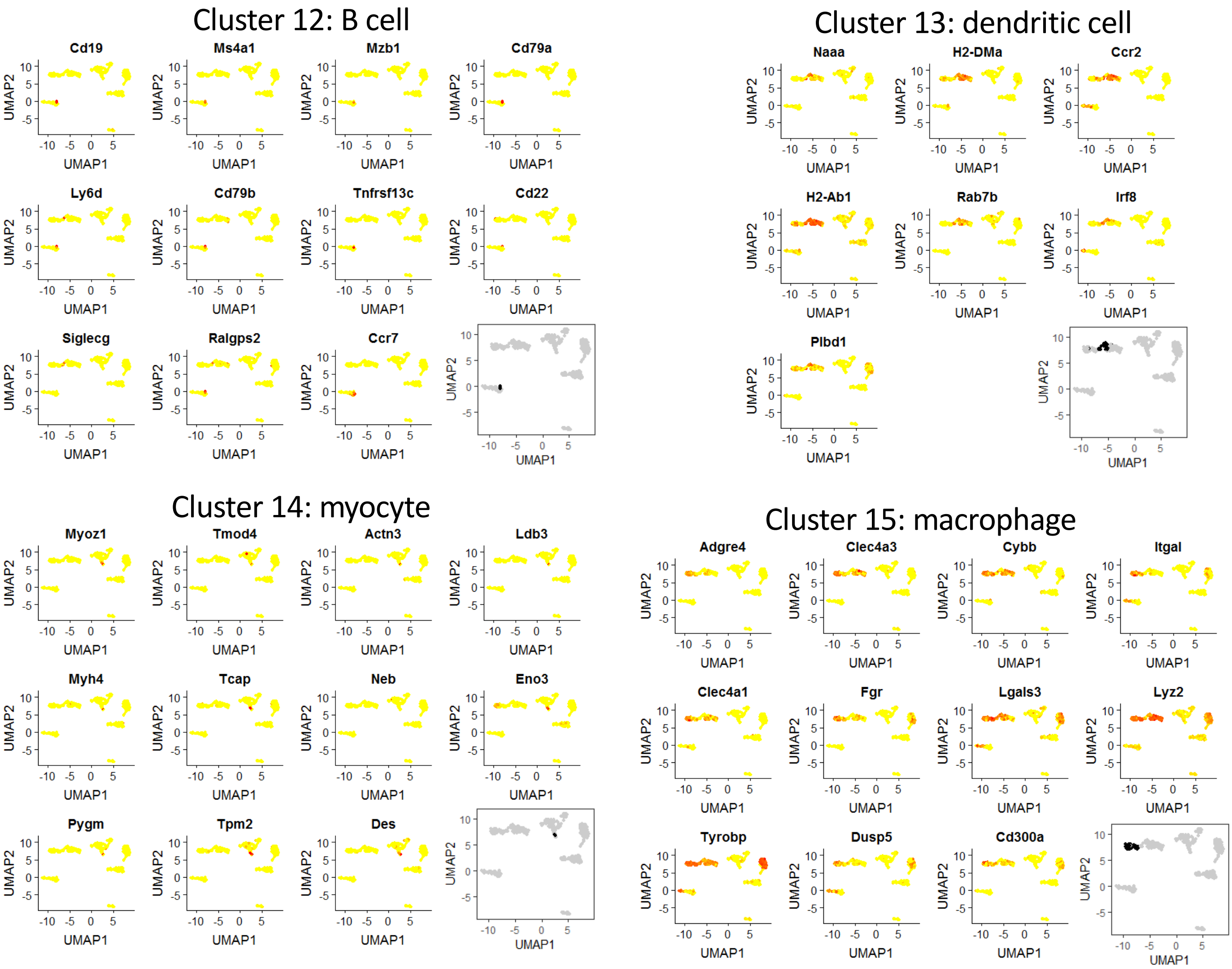

Supplementary Figure S11. (cont)

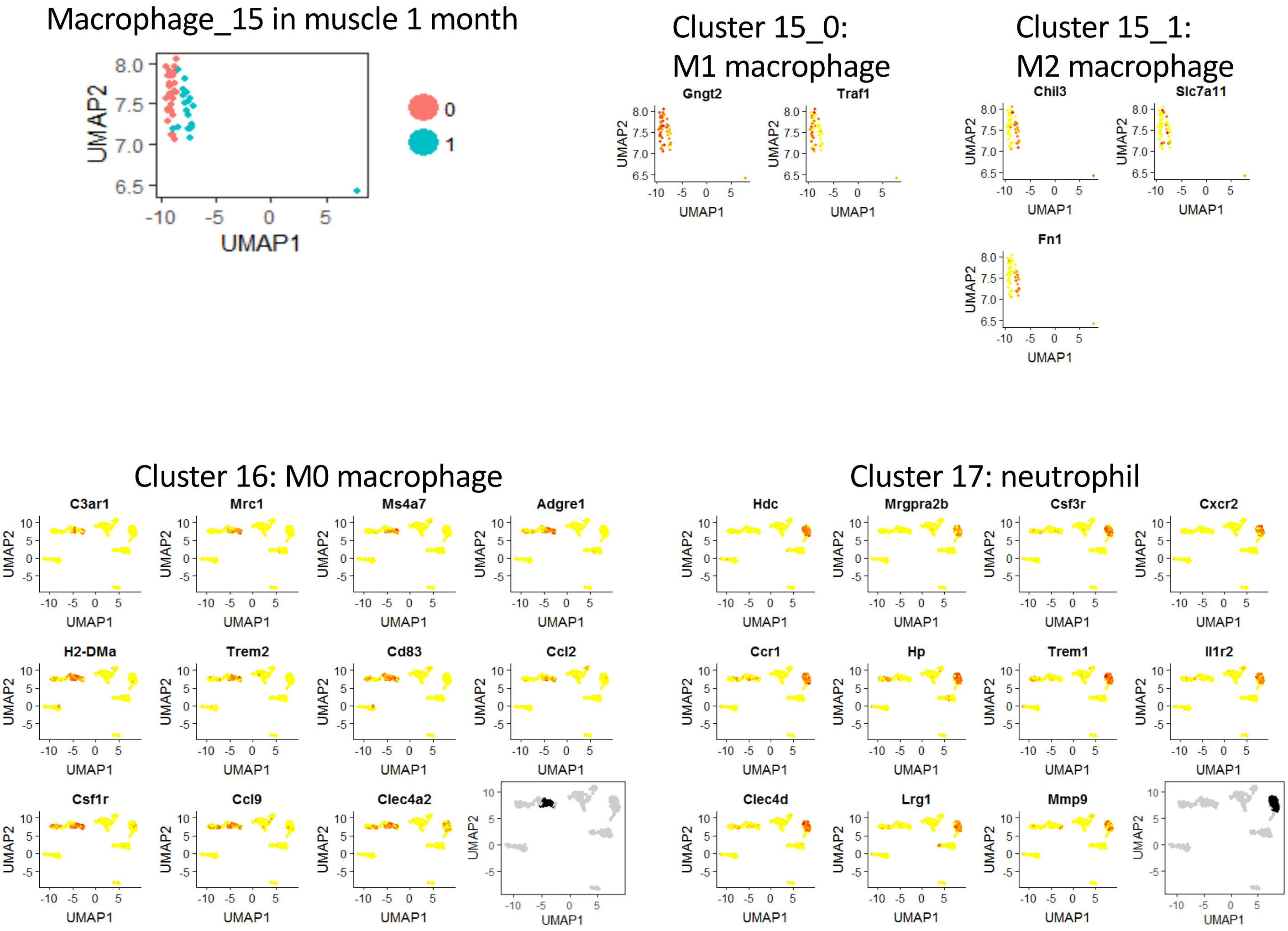

Supplementary Figure S11: UMAP projection of muscle cells 1 month post sepsis. As in Figure S10, cell type markers are shown for each cluster. Sub-clustering was done as follows: cluster 4 into endothelial cells and erythroid cells; cluster 6 into fibroblasts and mesenchymal stem cells; cluster 9 into T cells, NK cells, T memory cells; cluster 15 into M1 and M2 macrophages.

Supplementary Figure S12.

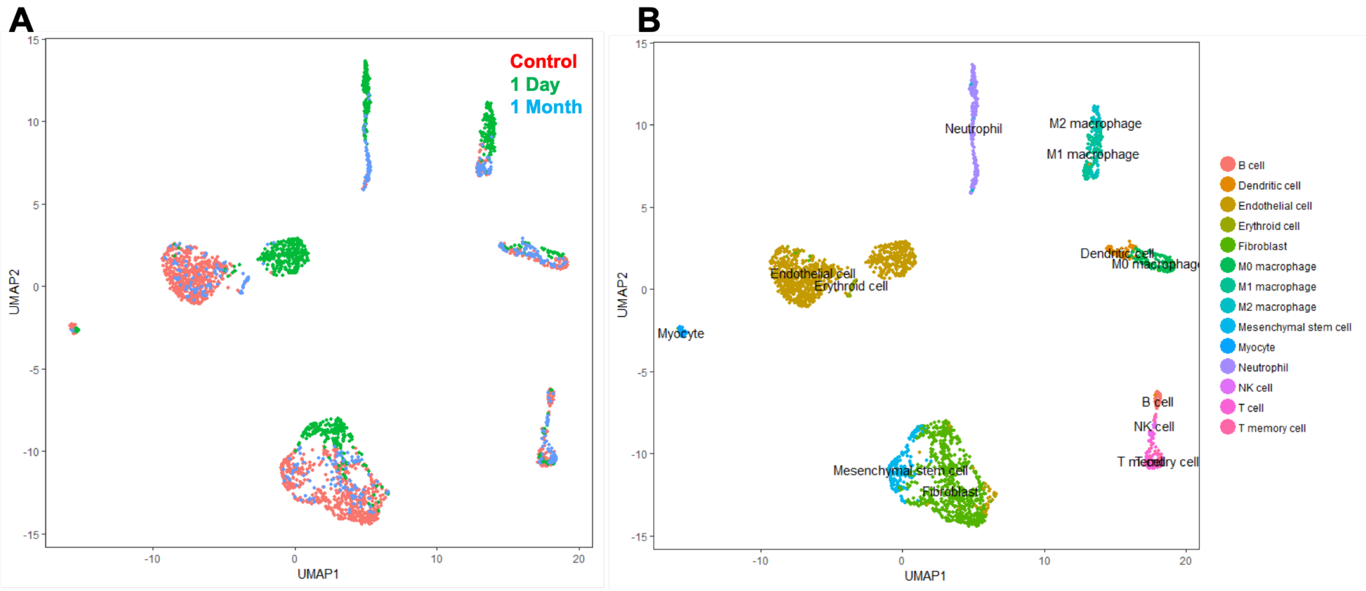

Supplementary Figure S12: Combined UMAP projections of post-sepsis skeletal muscle-resident cells. (A) Combined UMAP projection of control (red), 1 day post-sepsis (green), and 1 month post-sepsis (blue) muscle-derived cells. **(B)** Combined UMAP projection depicting the cell population identities of clusters depicted in (A).

Supplementary Figure S13.

Fat 1 day

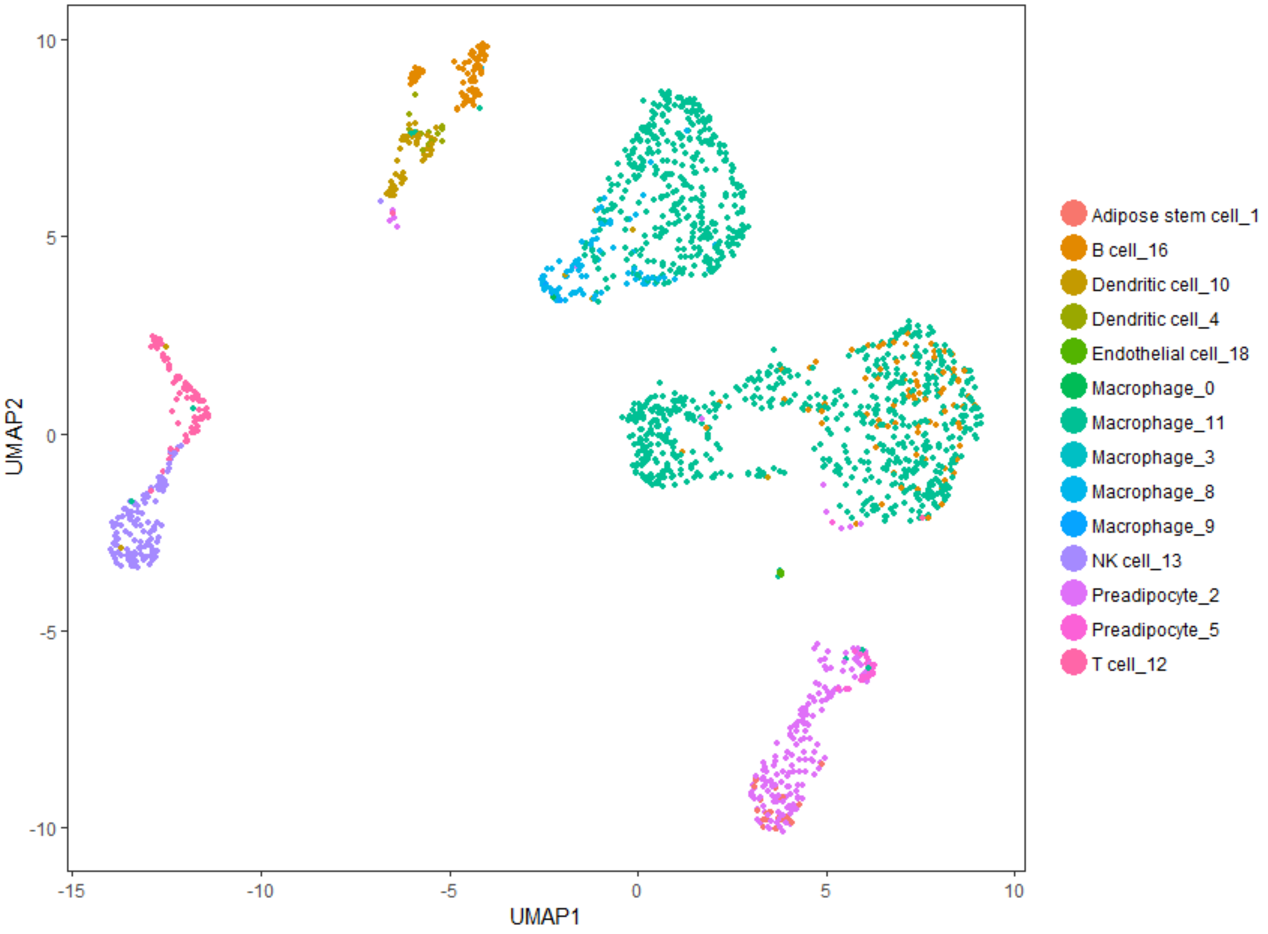

Cluster 0: macrophage

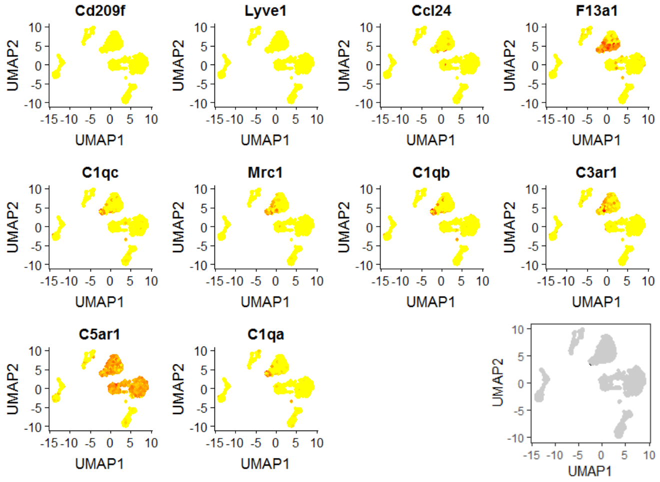

Cluster 1: adipose stem cell

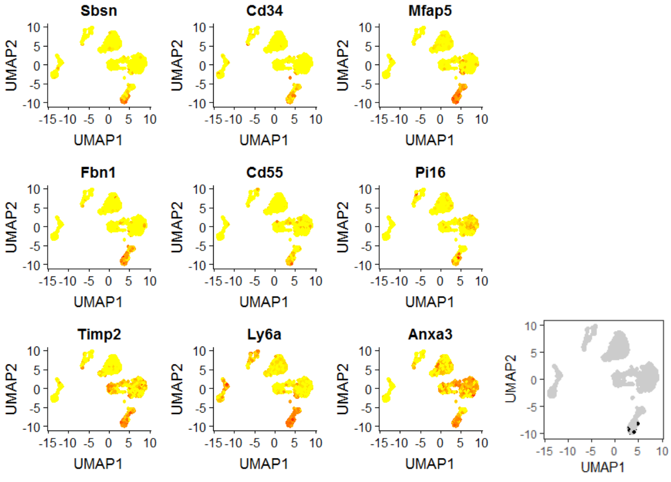

Supplementary Figure S13. (cont)

Cluster 2: preadipocyte

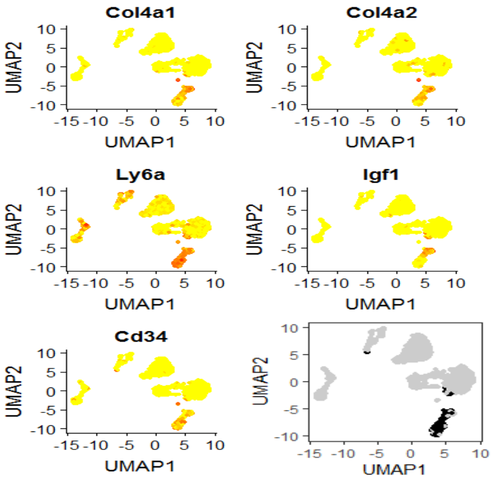

Cluster 3: macrophage

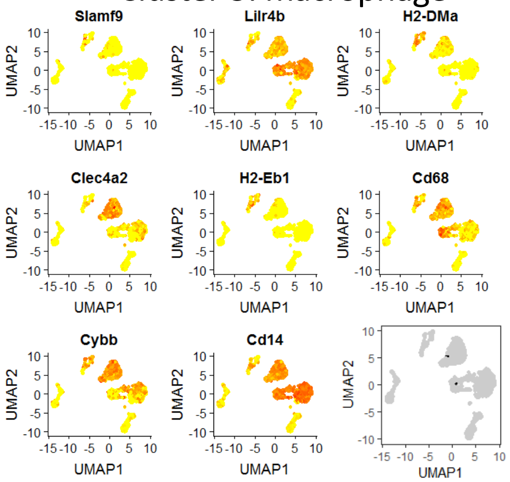

Cluster 4: dendritic cell

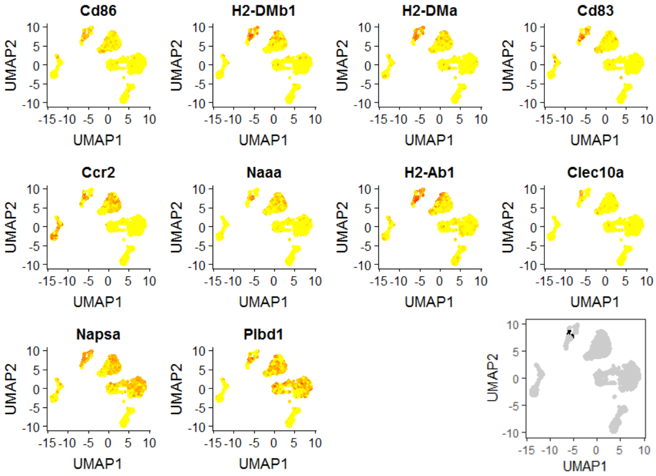

Cluster 5: preadipocyte

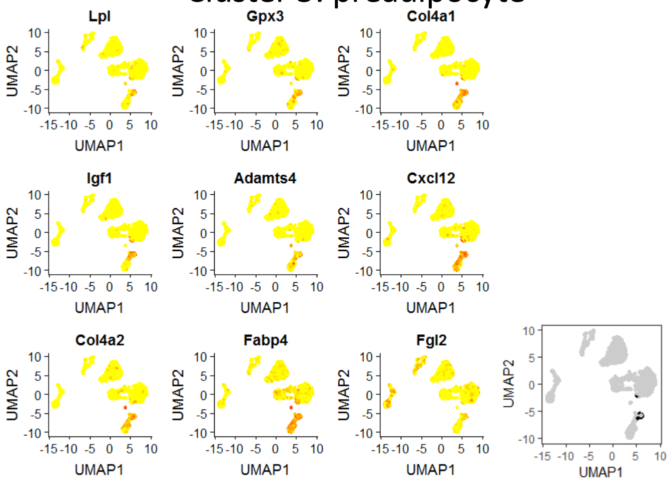

Cluster 8: macrophage

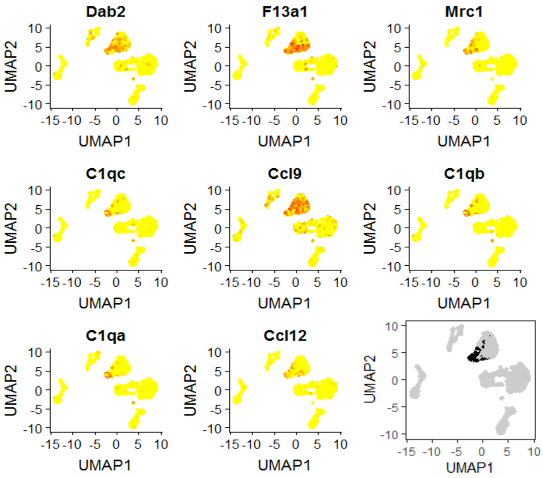

Cluster 9: macrophage

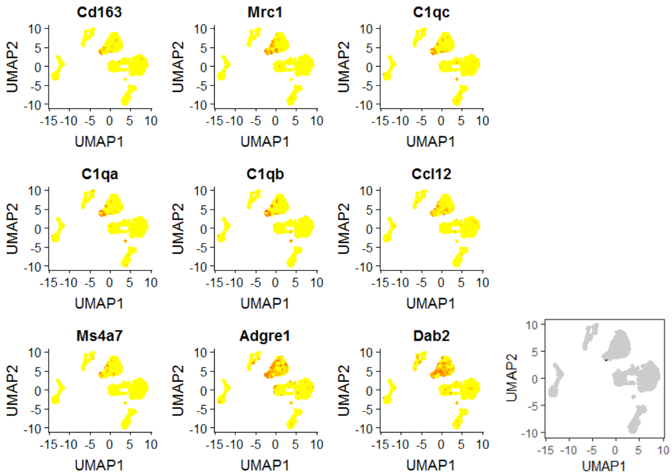

Supplementary Figure S13. (cont)

Cluster 10: dendritic cell

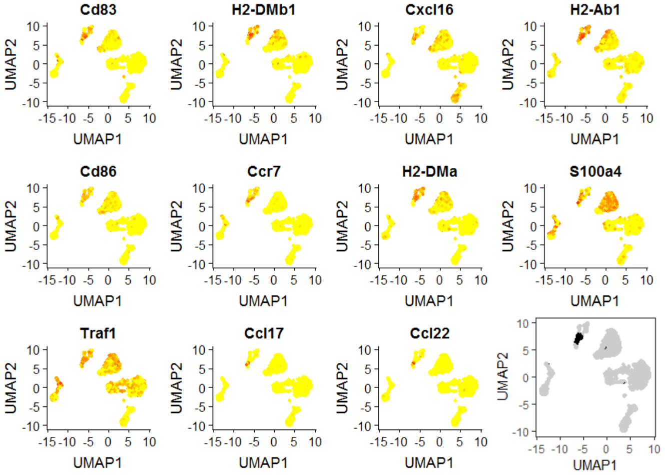

Cluster 12: T cell

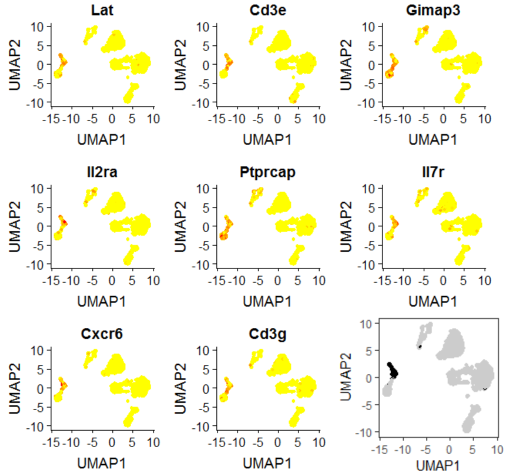

Cluster 13: NK cell

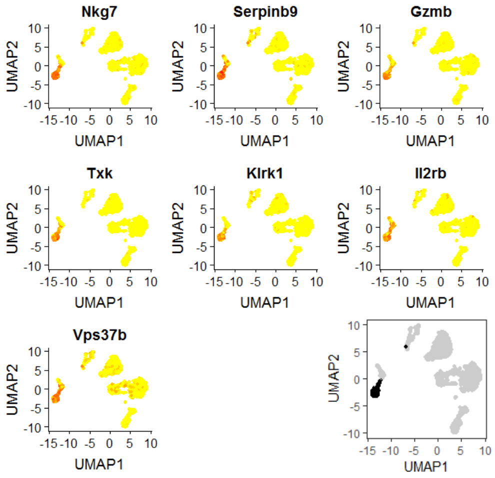

Cluster 18: endothelial cell

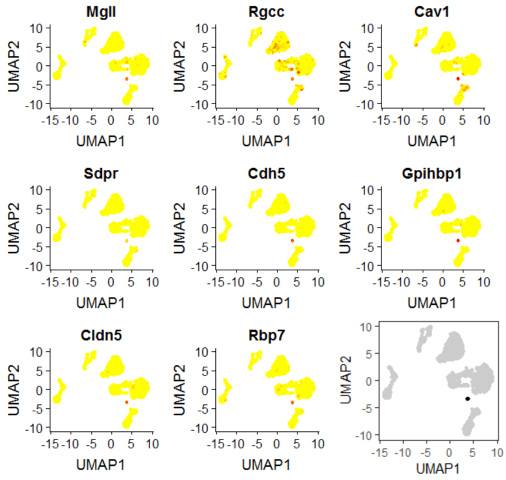

Cluster 11: Macrophage

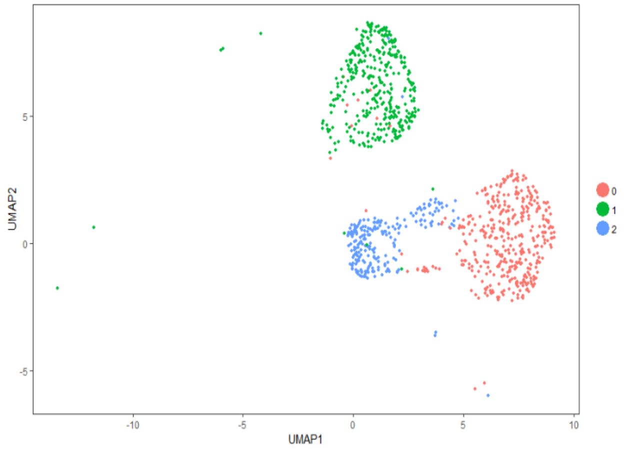

Supplementary Figure S13. (cont)

Cluster 11_0 & 11_2: neutrophil

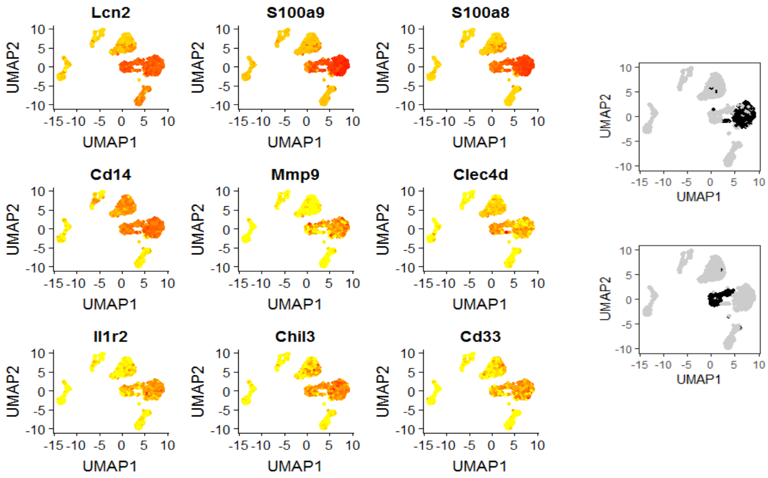

Cluster 11_1: macrophage

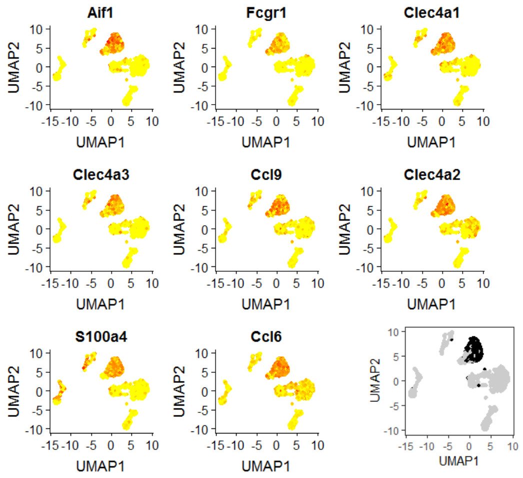

Cluster 16: B cell

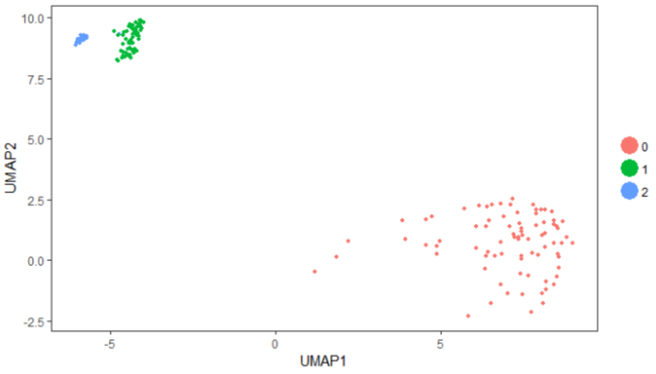

Cluster 16_0: neutrophil

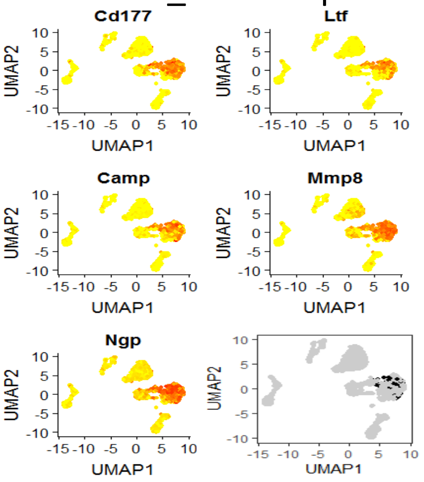

Cluster 16_1: B cell

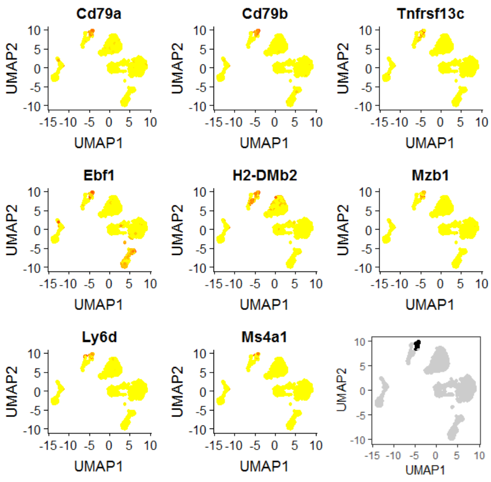

Cluster 16_2: plasmacytoid dendritic cell

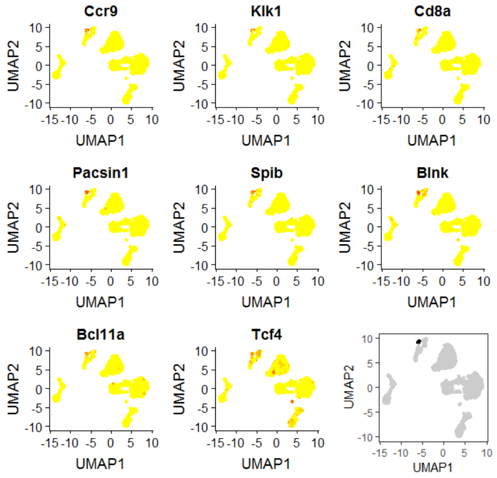

Supplementary Figure S13. (cont)

Supplementary Figure S13: UMAP projection of fat cells 1 day post sepsis. Cell type markers are shown for each cluster, like Figures S10-S11. Cluster 11 was sub-clustered into macrophages and neutrophils, and cluster 16 was sub-clustered into B cells, neutrophils, and plasmacytoid dendritic cells.

Supplementary Figure S14.

Fat 1 month

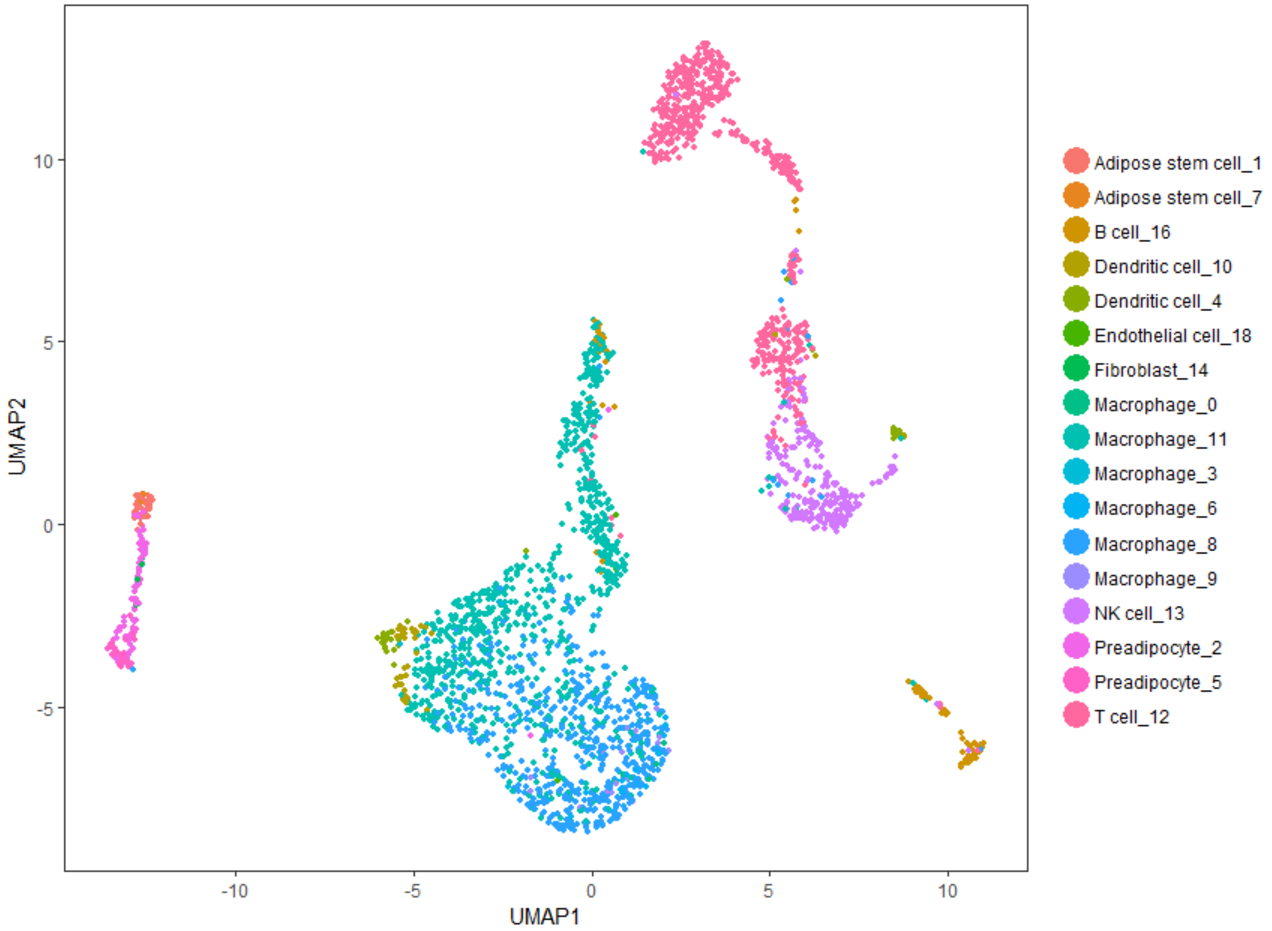

Cluster 0: macrophage

Cluster 2: preadipocyte

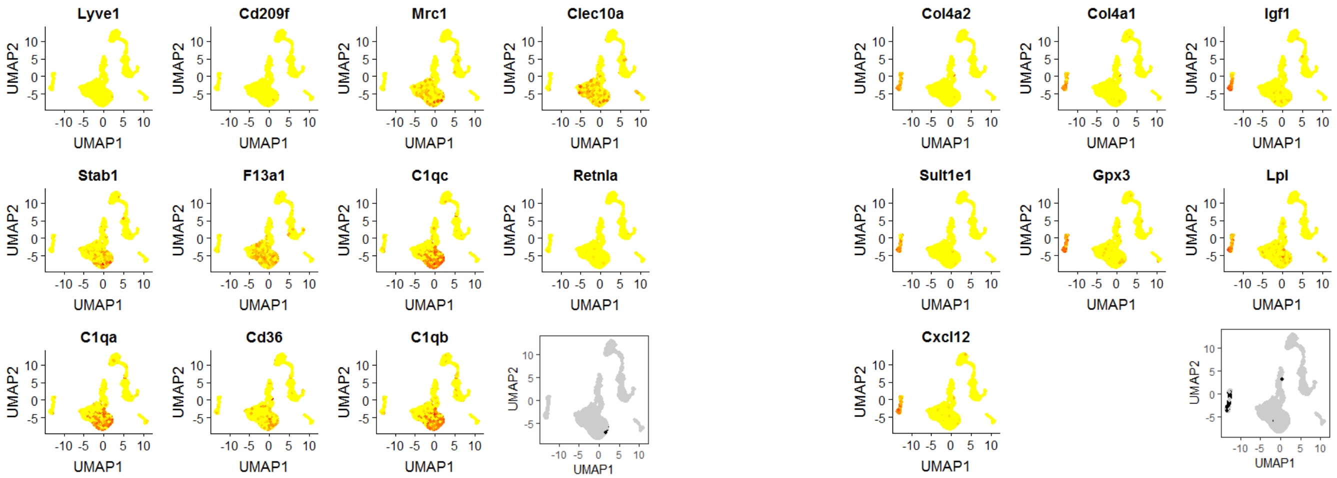

Supplementary Figure S14. (cont)

Cluster 1: adipose stem cell

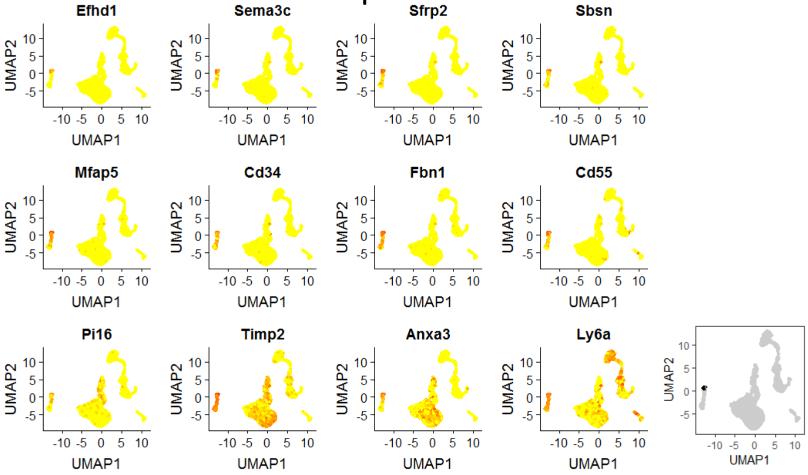

Cluster 3: macrophage

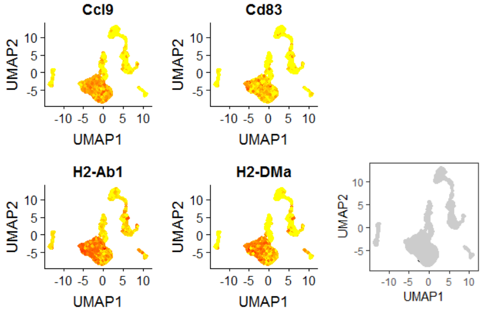

Cluster 4: dendritic cell

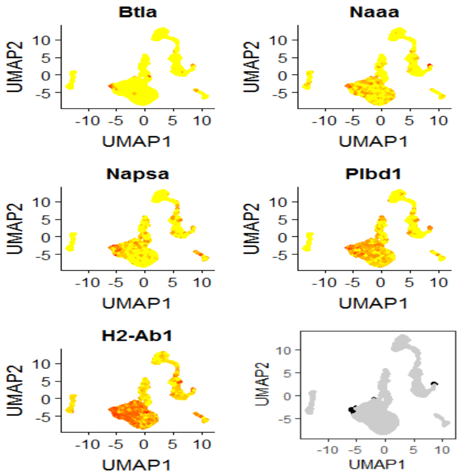

Cluster 5: preadipocyte

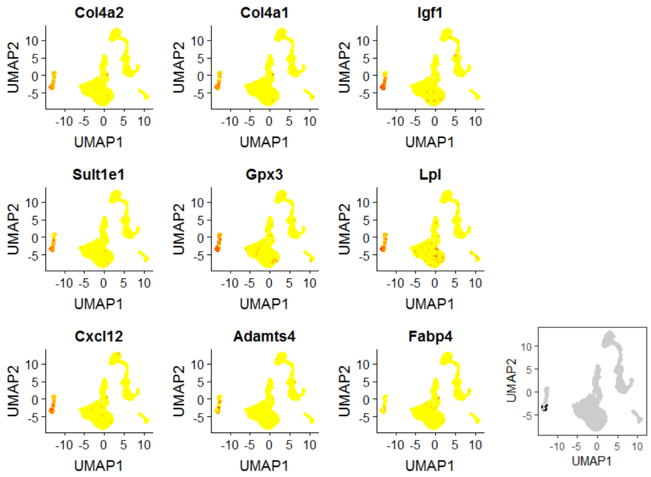

Cluster 6: macrophage

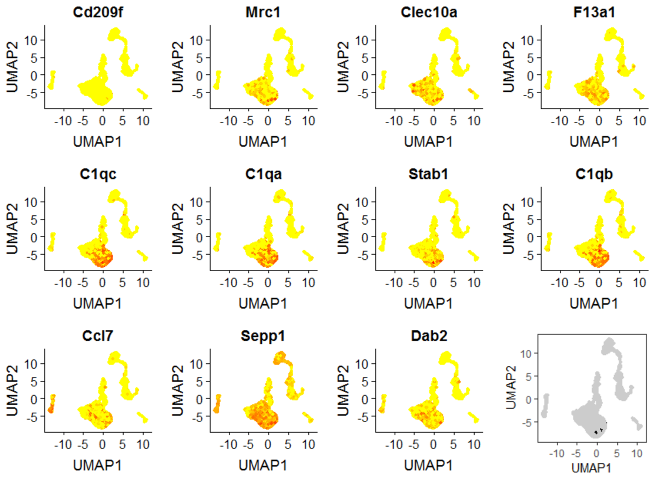

Supplementary Figure S14. (cont)

Cluster 7: adipose stem cell

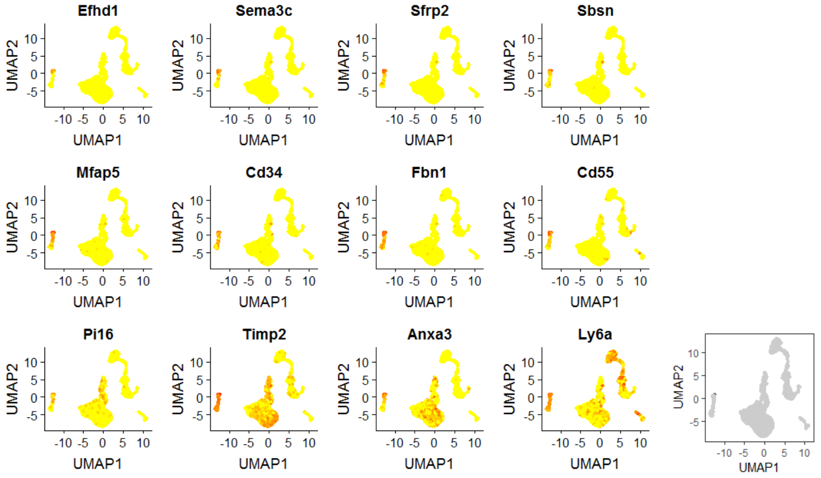

Cluster 8: macrophage

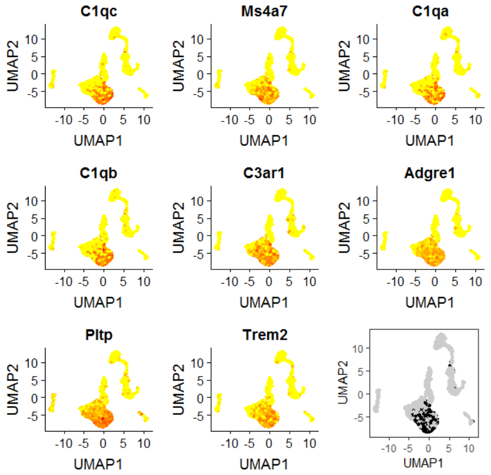

Cluster 9: macrophage

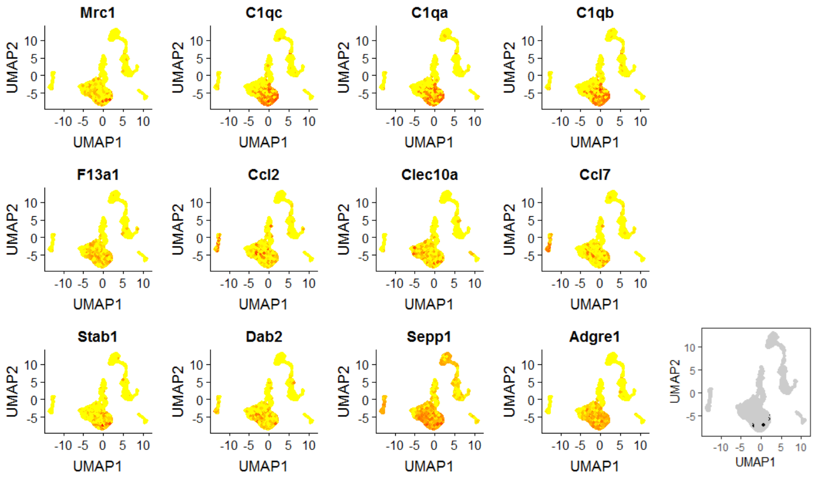

Cluster 10: dendritic cell

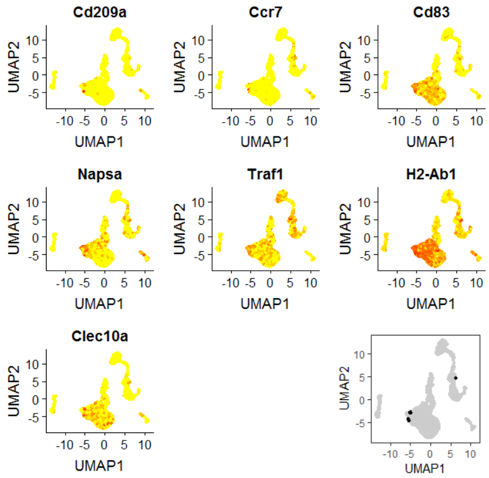

Cluster 11: Macrophage

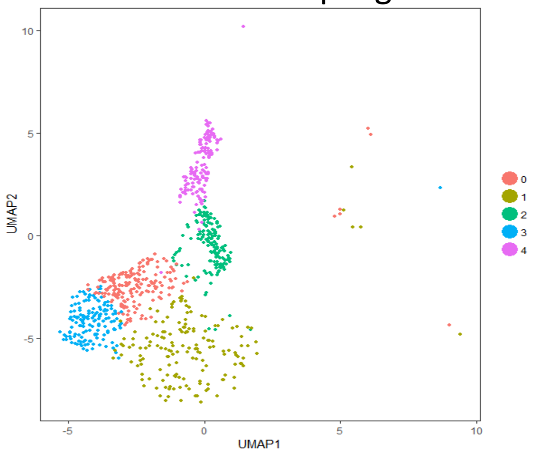

Cluster 11_0: macrophage

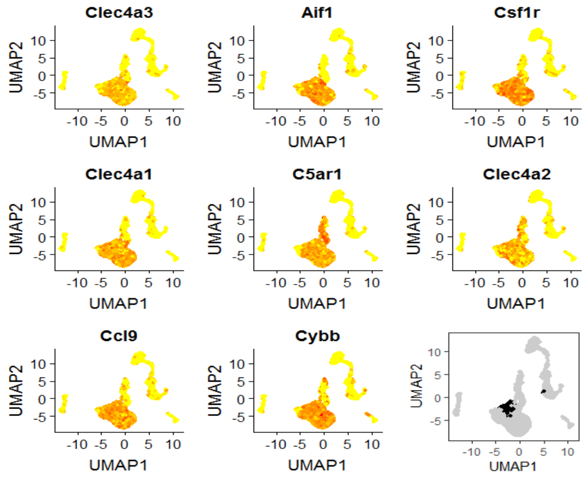

Supplementary Figure S14. (cont)

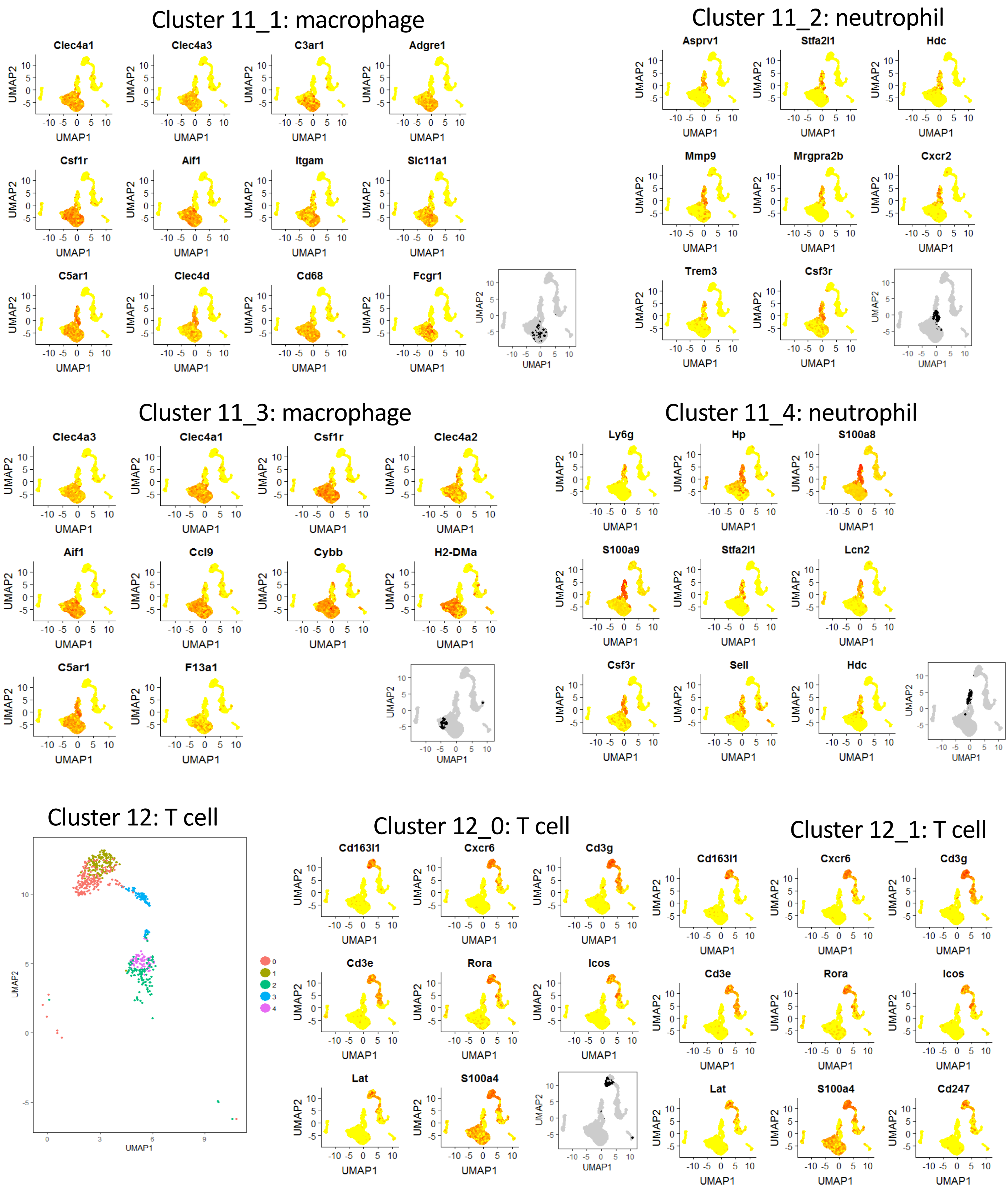

Supplementary Figure S14. (cont)

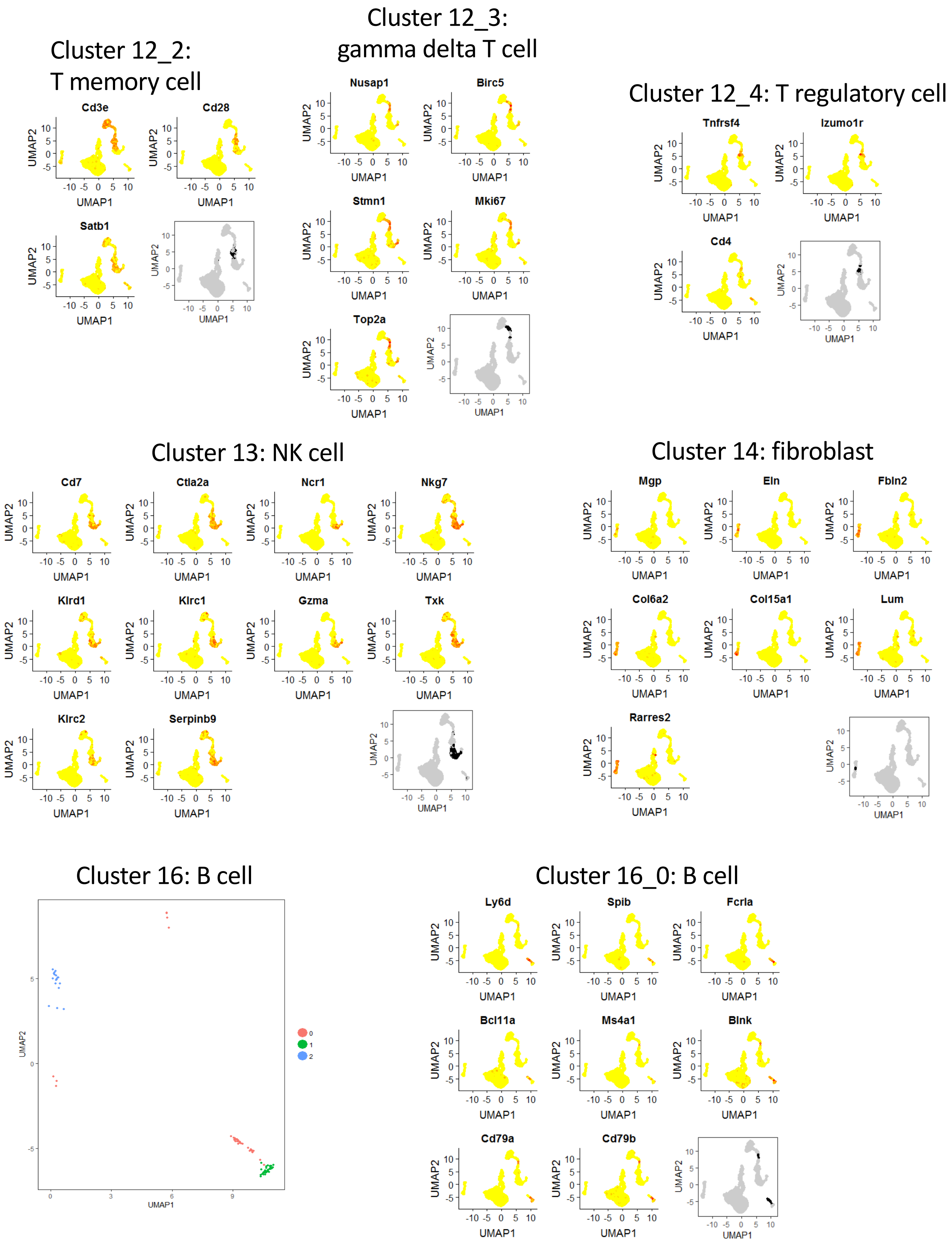

Supplementary Figure S14. (cont)

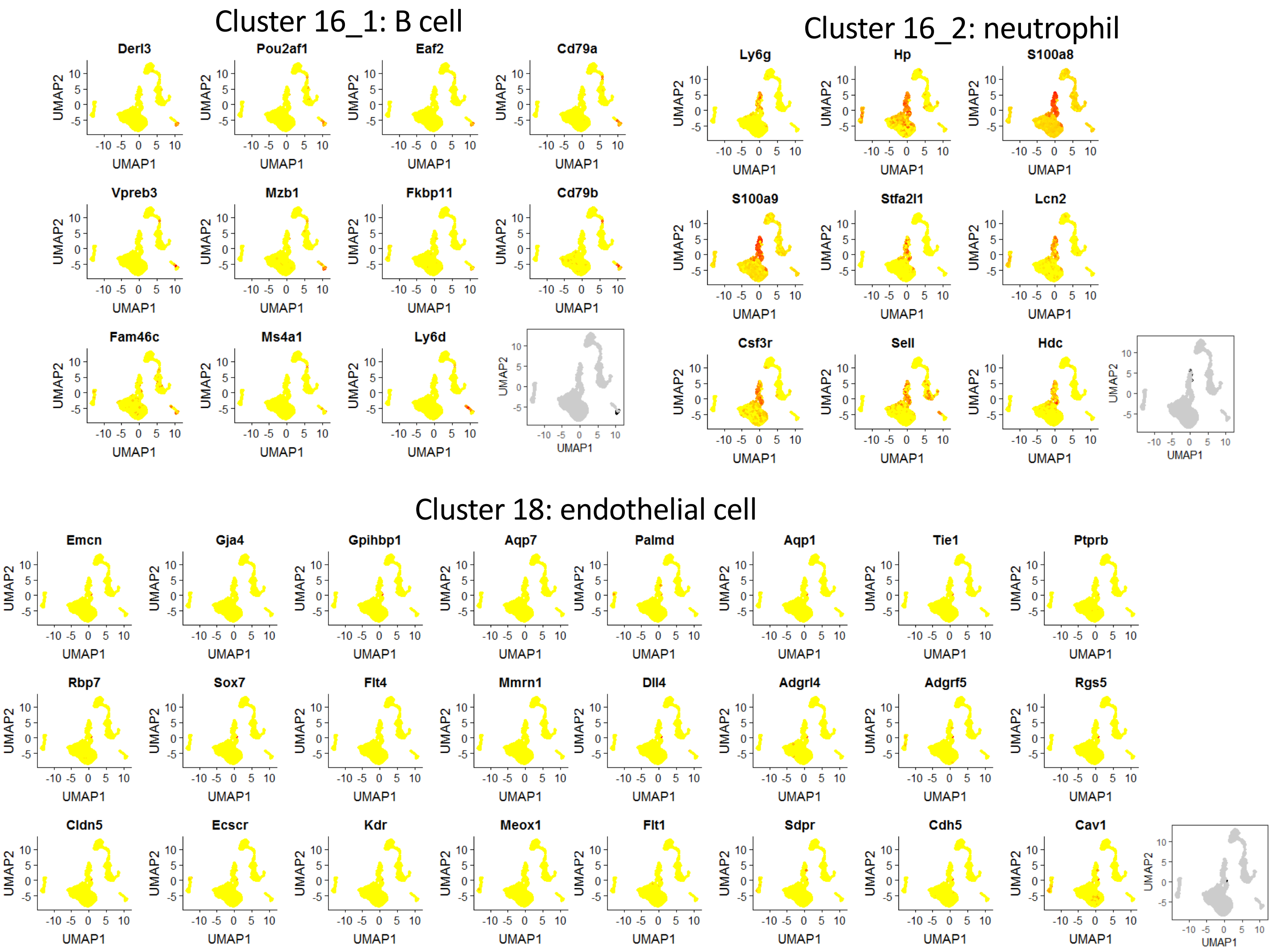

Supplementary Figure S14: UMAP projection of fat cells 1 month post sepsis. Cell type markers are shown for each cluster, like Figure S10-S11, S13. Sub-clustering was done as follows: cluster 11 into macrophages and neutrophils; cluster 12 into T cells, gamma delta T cells, and T memory cells, and T regulatory cells; cluster 16 into B cells and neutrophils.

Supplementary Figure S15.

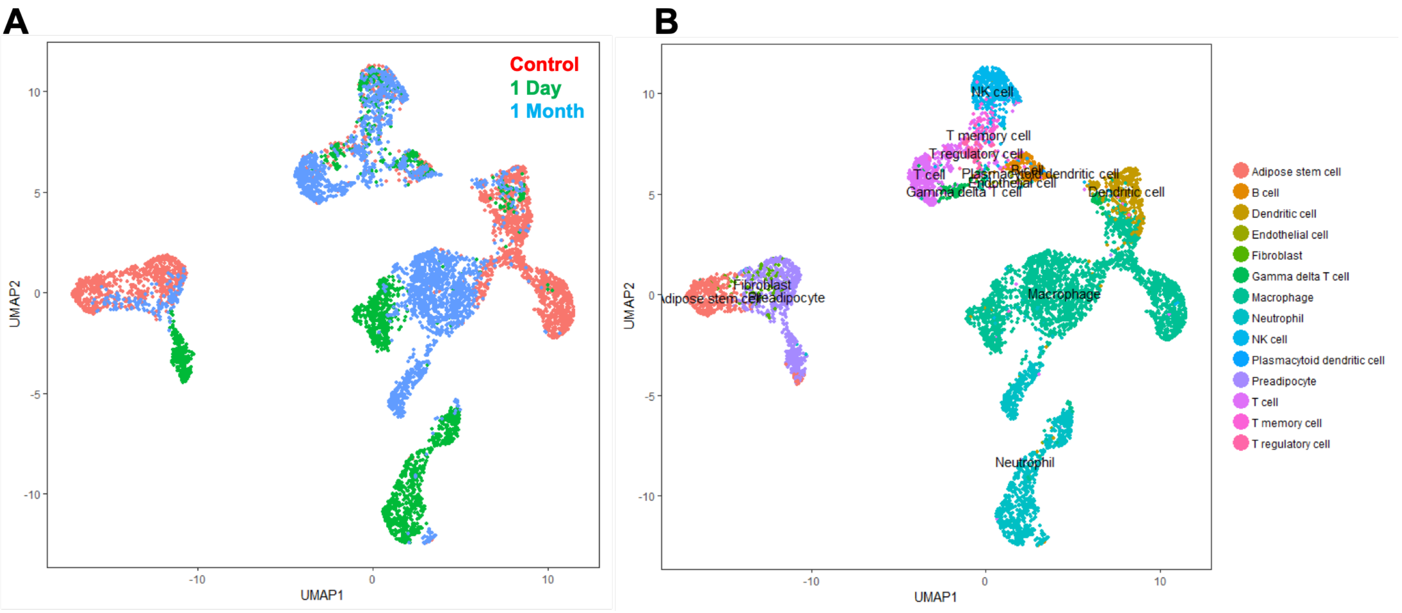

Supplementary Figure S15: Comparative analysis of post-sepsis adipose-resident cell population abundance. (A) Combined UMAP projection of control (red), 1 day post-sepsis (green), and 1 month post-sepsis (blue) adipose-derived cells. **(B)** Combined UMAP projection depicting the cell population identities of clusters depicted in (A).

Supplementary Figure S16.

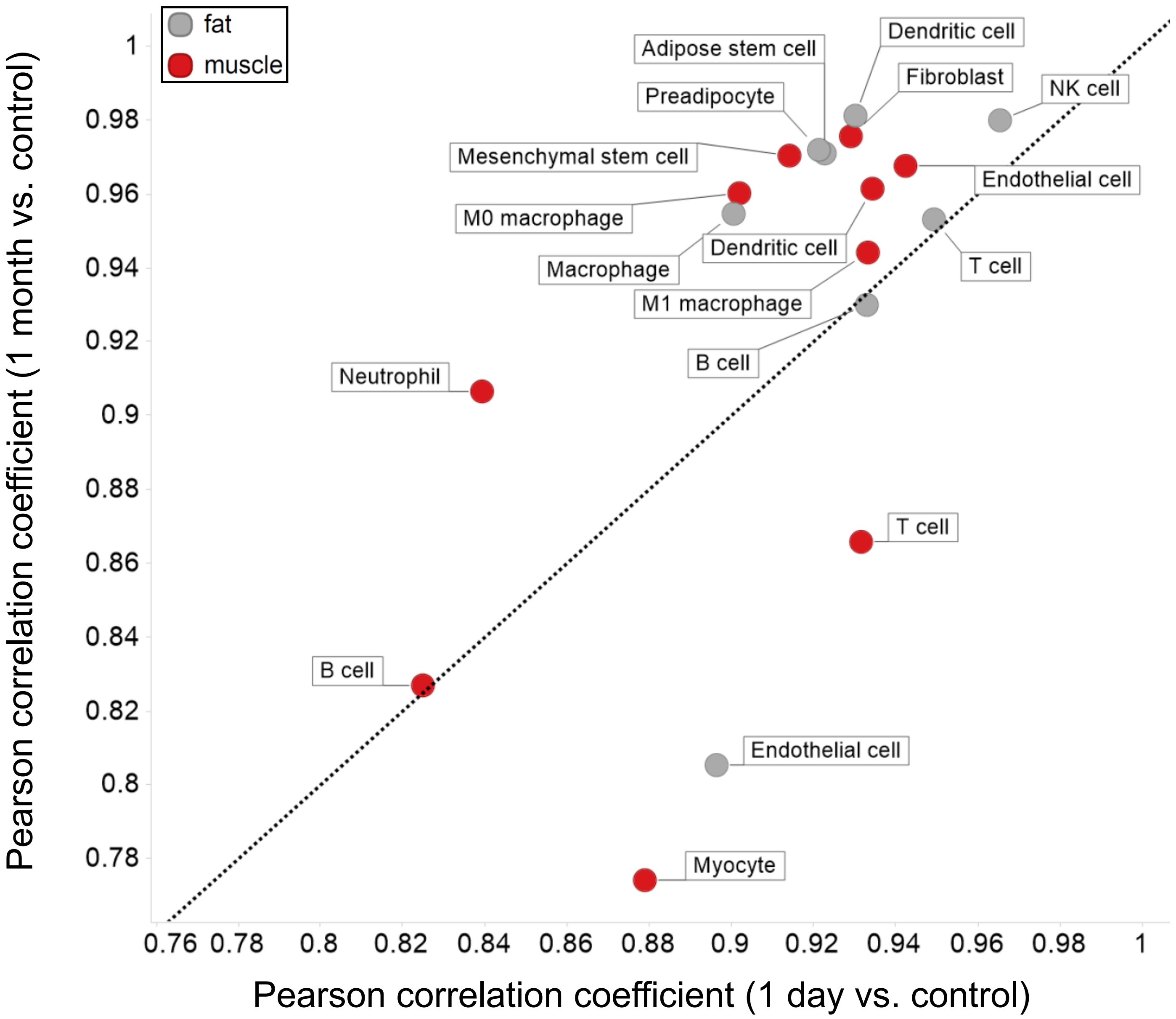

Supplementary Figure S16: A combined scatter plot depicting control-normalized Pearson correlation coefficients of 1 day and 1 month post-sepsis cell types in muscle and fat. Gray=fat-derived population, red=muscle-derived population.

Supplementary Figure S17.

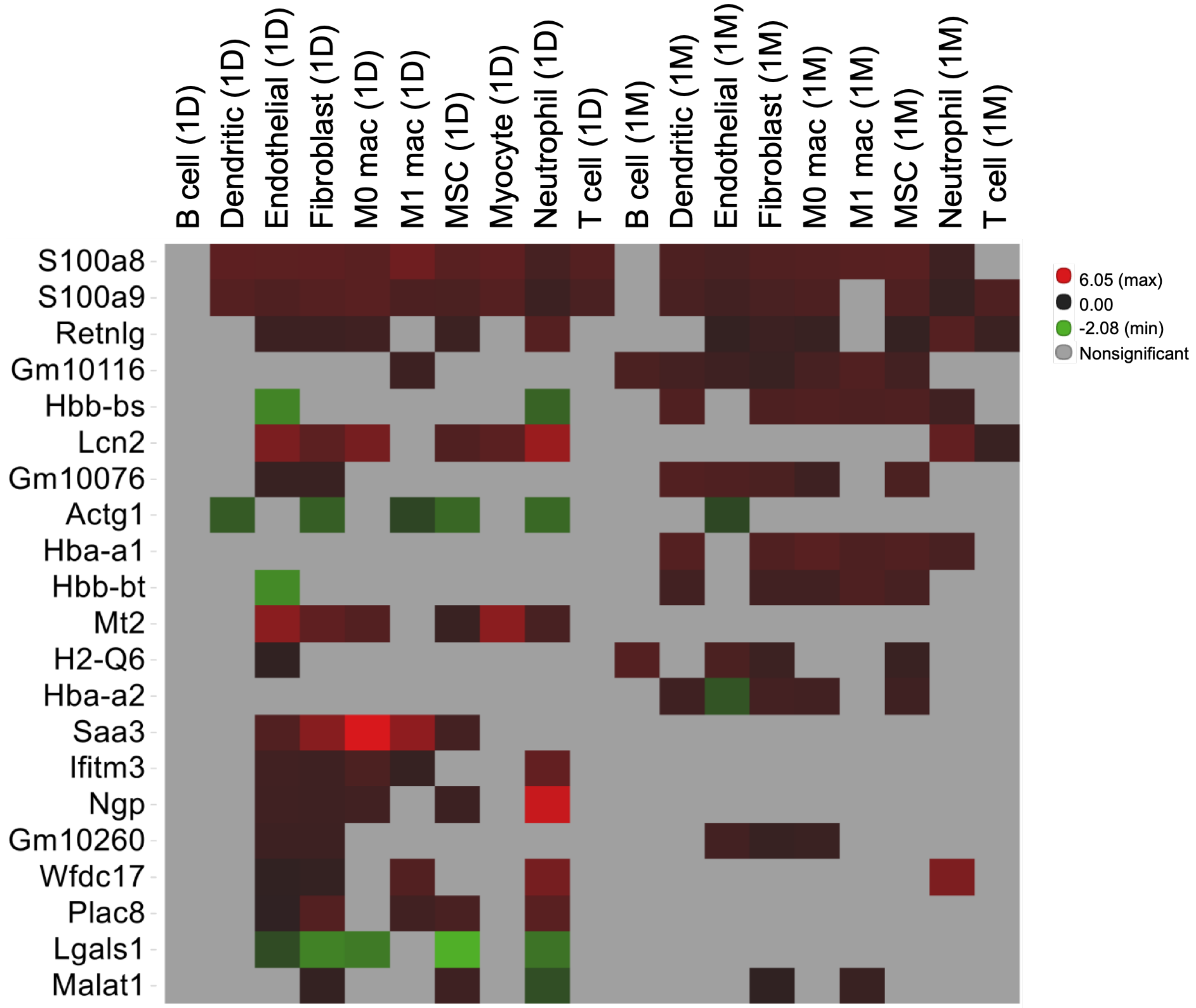

Supplementary Figure S17: Top differently expressed (DE) genes identified in multiple cell types in muscle. A heatmap depicting top DE genes identified in at least 5 comparisons in muscle. Log fold changes compared to the same cell type in control were shown with colors. Red=up-regulated, green=down-regulated, gray=no significant difference.

Supplementary Figure S18.

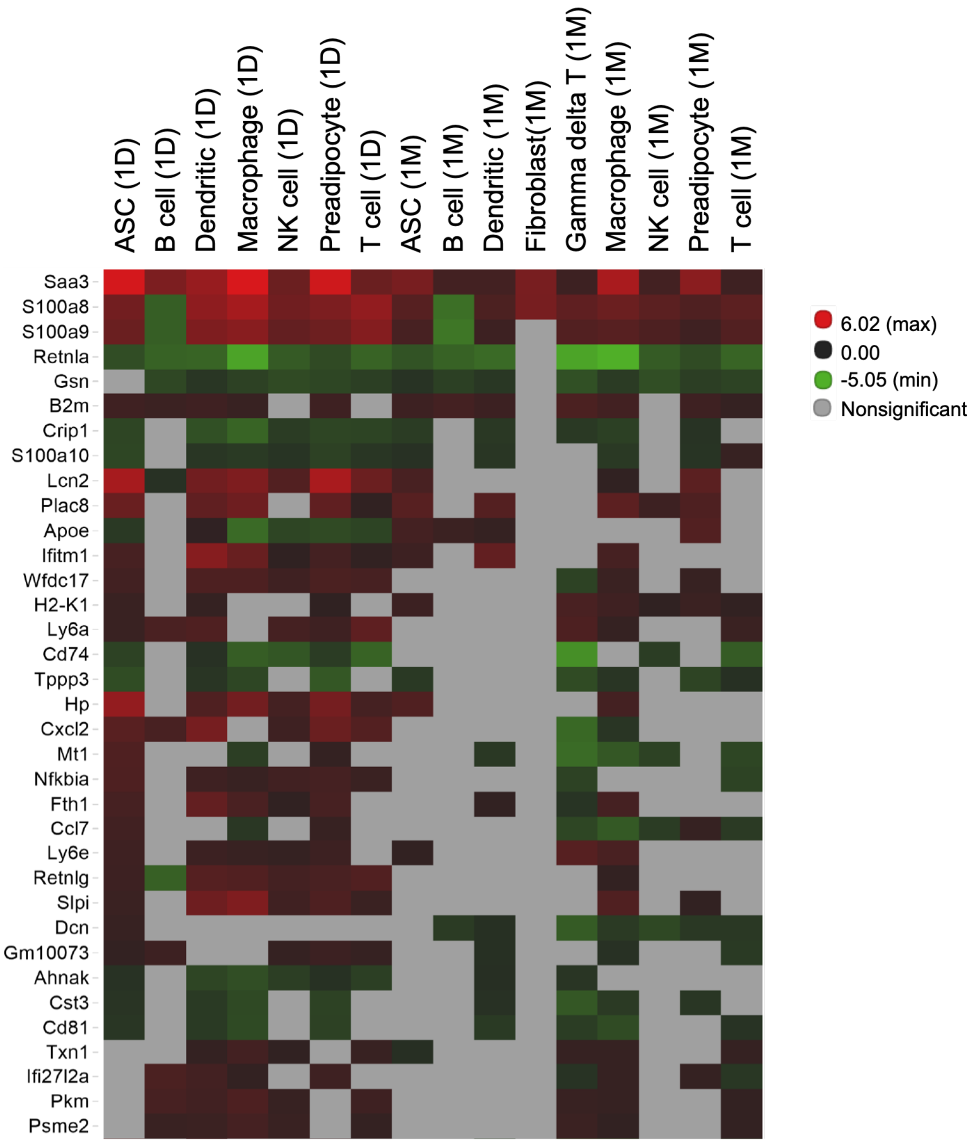

Supplementary Figure S18: Top differently expressed (DE) genes identified in multiple cell types in adipose tissue. A heatmap depicting top DE genes identified at least 8 comparisons in adipose tissue. Log fold changes compared to the same cell type in control were shown with colors. Red=up-regulated, green=down-regulated, gray=no significant difference.
